# Multi-omics study reveals that statin therapy is associated with restoration of gut microbiota homeostasis and improvement in outcomes in patients with acute coronary syndrome

**DOI:** 10.7150/thno.55946

**Published:** 2021-03-31

**Authors:** Xiaomin Hu, Hanyu Li, Xinyue Zhao, Ruilin Zhou, Honghong Liu, Yueshen Sun, Yue Fan, Yanan Shi, Shanshan Qiao, Shuangjiang Liu, Hongwei Liu, Shuyang Zhang

**Affiliations:** 1 Department of Medical Research Center, State Key Laboratory of Complex Severe and Rare Diseases, Peking Union Medical College Hospital, Chinese Academy of Medical Science & Peking Union Medical College, 100730, Beijing, China.; 2 Department of Cardiology, Peking Union Medical College Hospital, Chinese Academy of Medical Science & Peking Union Medical College, 100730, Beijing, China.; 3 State Key Laboratory of Mycology, Institute of Microbiology, Chinese Academy of Sciences, 100101, Beijing, China; Savaid Medical School, University of Chinese Academy of Sciences, 100049, Beijing, China.; 4 State Key Laboratory of Microbial Resources, Institute of Microbiology, Chinese Academy of Sciences, 100101, Beijing, China.

**Keywords:** statins, acute coronary syndrome, outcome, microbiome, metabolomics

## Abstract

**Rationale:** Prior chronic treatment with statins has been shown to be associated with more favorable outcomes in patients with acute coronary syndrome (ACS). Specific changes in the gut microbiota and microbial metabolites have been shown to influence the progression of coronary artery disease. However, the critical microbial and metabolomic changes associated with the cardiovascular protective effects of statins in ACS remain elusive.

**Methods:** In the present study, we performed 16S rRNA sequencing and serum metabolomic analysis in 36 ACS patients who had received chronic statin treatment, 67 ACS patients who had not, and 30 healthy volunteers. A follow-up study was conducted. Metagenomic functional prediction of important bacterial taxa was achieved using PICRUSt2.

**Results**: Statins modulated the gut microbiome of ACS patients towards a healthier status, i.e., reducing potentially pathogenic bacteria such as* Parabacteroides merdae* but increasing beneficial bacteria such as *Bifidobacterium longum subsp. longum*, *Anaerostipes hadrus* and *Ruminococcus obeum*. Moreover, prior chronic statin therapy was associated with improved outcome in ACS patients. Multi-omics analysis revealed that specific changes in bacterial taxa were associated with disease severity or outcomes either directly or by mediating metabolites such as fatty acids and prenol lipids. Finally, we discovered that important taxa associated with statins were correlated with fatty acid- and isoprenoid-related pathways that were predicted by PICRUSt2.

**Conclusions:** Our study suggests that statin treatment might benefit ACS patients by modulating the composition and function of the gut microbiome, which might result in improved circulating metabolites and reduced metabolic risk. Our findings provide new insights for understanding the heterogenic roles of statins in ACS patients through host gut microbiota metabolic interactions.

## Introduction

Due to the improvements in guideline-directed medical therapy (GDMT) and interventions, the incidence and mortality rates of acute coronary syndromes (ACS) are diminishing 1. Nonetheless, ACS remains one of the major causes of premature death in adults, especially in developed countries 2. ACS refers to any group of clinical symptoms compatible with acute myocardial ischemia 3. Based on the degree of coronary artery occlusion as well as myocardial ischemic injury, ACS can be further divided into unstable angina (UA), non-ST elevation myocardial infarction (NSTEMI) and ST elevation myocardial infarction (STEMI). It is estimated that >780,000 persons will experience ACS each year in the United States 4. The 1-year postdischarge mortality rates are approximately 8.4% for patients with STEMI and 18.7% for patients with NSTEMI 5.

Lipid-lowering treatment is an indispensable part of GDMT for patients with coronary artery disease (CAD). In particular, the initiation of statins is recommended as a class IA therapy for secondary prevention in patients with CAD and other atherosclerotic vascular diseases 6, 7. Long-term treatment with statins could reduce the risk of all-cause mortality in coronary heart disease (CHD) patients 8. In particular, prior chronic treatment with statins is associated with a reduced prevalence of ruptured plaques as well as favorable outcomes in patients presenting with ACS 9-11. Some side effects of statins have been reported, e.g., rhabdomyolysis, hepatoxicity and a higher risk of new-onset diabetes mellitus. However, the cardiovascular protective effects of statins are considered to outweigh the possible risks 12, 13. Statins block cholesterol biosynthesis in a competitive way by inhibiting 3-hydroxy-3-methyl-glutaryl-CoA (HMG-CoA) reductase, which catalyzes the rate-limiting step in the synthesis of the precursor for cholesterol. In addition, statins can promote the removal of low-density lipoprotein cholesterol (LDL-C) from the blood by inducing the expression of LDL receptor (LDL-R) 14. Moreover, statins have pleiotropic effects, such as immunomodulatory activities 14, 15.

Currently, significant interest has been focused on the roles of gut dysbiosis in CAD. Based on our previous study, the bacterial co-abundance group (CAG), which includes *Veillonella*, *Haemophilus* and *Klebsiella*, and specific metabolites, such as taurine, sphingolipid and ceramide, exhibit characteristic changes with the severity of CAD 16. Moreover, gut microbe-derived metabolites, including trimethylamine-N-oxide (TMAO) and phenylacetyl glutamine (PAGln), have been shown to be involved in atherosclerosis and thrombosis, thus contributing to cardiovascular diseases 17-19. Since statins are widely used as first-line agents for the treatment of dyslipidemia and atherosclerosis, multiple studies have focused on the relationship between statin use and gut microbiota variation. Several animal experiments demonstrated that statin use could lead to alterations in the gut microbiota as well as intestinal metabolites such as bile acid (BA) pool and short chain fatty acids (SCFAs) 20-23. On the other hand, the composition of the host gut microbiota has also been revealed to influence the bioavailability of statins *in vivo* 24-27. However, to the best of our knowledge, no published study has focused on statin-associated gut microbiota and serum metabolomic alterations or their associations with clinical phenotypes in ACS.

To address the question above, we recruited a total of 133 subjects at Peking Union Medical College Hospital (PUMCH) and divided them into a control group, an ACS group (patients who had not taken any statins within 4 weeks before recruitment) and an ACS-statins group (patients who had been receiving standard statin medication for at least 4 weeks until recruitment). Detailed clinical data were collected, and postdischarge follow-up was conducted. Furthermore, 16S rRNA sequencing and liquid chromatography-mass spectrometry (LC-MS) were applied. The objectives of this study were (1) to reveal the variation in the gut microbiota and metabolic profile that may be associated with statin use in ACS and (2) to investigate the correlations between the gut microbiome, serum metabolome and clinical outcomes in ACS.

## Methods

### Study design and population

We consecutively recruited patients who were hospitalized for coronary angiography at PUMCH. The grouping criteria for patients were as follows: (1) male or female patients who exhibited ≥50% stenosis in at least one main coronary artery based on coronary angiography images and (2) the main admitting diagnosis of patients was UA, NSTEMI or STEMI. The detailed diagnostic criteria are summarized in the supplementary methods. Coronary atherosclerotic burden was assessed using the Gensini score by two professional cardiologists as described in our previous publication 16. In addition, we recruited healthy volunteers who met the following criteria: (1) exhibited no CAD-related clinical symptoms or signs or exhibited negative results upon coronary artery CT or coronary angiography and (2) had not taken any statins within 4 weeks before recruitment. Subjects with one or more of the following conditions were excluded from this study: (1) received antibiotic treatment for more than 3 consecutive days within 3 months before enrollment; (2) had gastrointestinal diseases or received gastrointestinal surgery within 1 year; and (3) had any malignant tumors, autoimmune disorders, infectious diseases or severe renal dysfunction (creatine >3.0 mg/dl).

Patients enrolled in this study were further divided into two groups based on their medication: patients who had taken long-term (≥4 weeks) and standard oral statins before recruitment were included in the ACS-statins group, and patients who had not taken any statins within 4 weeks before recruitment were included in the ACS group. For each subject, fasting peripheral venous blood and stool samples were collected in the morning the day after admission. The preparation and storage of serum and stool samples, as well as procedures for collecting the clinical information on each subject, were described in our previous publication 16. Statistical analysis of baseline characteristics is described in the supplementary methods. This study was executed conforming to the principles of the Declaration of Helsinki. All subjects provided written, informed consent for participation in the study.

### Follow-up study

Postdischarge follow-up was conducted when patients were reviewed in the clinic, or through telephone interviews with patients/their close family members. The composite endpoint of this study consisted of all-cause mortality and/or reoccurrence of ACS and/or stroke and/or readmission for cardiac causes. The identification of new hospitalization and/or all-cause mortality was based on the electronic medical record system of PUMCH or interviews with the patient (or a family member) in cases of events outside of PUMCH. Binary logistic regression analysis was employed to explore the relationship between statin therapy before enrollment and the outcome of patients with ACS after adjusting for potential confounding factors. The statistical analysis was performed using the SPSS statistics software (v24.0, SPSS Inc., Chicago, IL, USA). The results of binary logistic regression were visualized as forest plots using the R package ggplot2 28.

### 16S rRNA sequencing and processing

The procedures for total fecal DNA extraction, amplification and sequencing of the V3-V4 region of the 16S rRNA genes as well as the construction of the sequencing library were illustrated in previous publications 16, 29-31. The downstream amplicon bioinformatic analyses were performed with EasyAmplicon v1.0 32. Dereplication was performed using the *-derep_fullength* command of VSEARCH (v2.15) 33. Then, the nonredundant sequences were denoised into amplicon sequence variants (ASVs) via the *-unoise3* command of USEARCH (v10.0) 34. The feature (ASV) table was created with *vsearch --usearch_global*. Taxonomic classification of ASVs was achieved using the sintax algorithm of USEARCH based on the Ribosomal Database Project (RDP) training set v16 35. The sequences of all samples were rarefied to 10,048 for the downstream diversity analysis. Alpha diversity analysis was carried out using the vegan package (v2.5-6) in R v4.0.2 36. Rarefaction curves were generated using *usearch -alpha_div_rare*. Differences in Shannon's index and the Chao1 index between groups were evaluated using Tukey's HSD test. A phylogenetic tree was constructed based on the ASVs using *usearch -cluster_agg*. The weighted UniFrac distance matrix was generated using *usearch -beta_div*. Beta diversity calculations were performed by principal coordinate analysis (PCoA), and the Adonis test was applied to test for significant differences between groups. The R package ggplot2 was used to visualize the results of the diversity analyses. Since dietary intake is an important host factor affecting the gut microbiota, we collected information on dietary frequency from subjects and calculated their baseline nutrient intake (detailed methods were described in our previous study 16). The Adonis test was utilized to calculate the explanation rate of each dietary intake factor based on 99 permutations.

### Analysis of the taxonomic composition and prediction of flora phenotype

The taxonomic composition of each group was visualized as a stacked bar plot at the phylum level and as a chord plot at the genus level with the ggplot2 package. For difference comparisons, edgeR was utilized to identify significantly differential features between groups 37 and the Benjamini-Hochberg method was used to control the FDR. To obtain functional predictions based on the 16S rRNA sequences, the taxonomic classification of sequences based on the Greengenes database was performed using *usearch -otutab* 38. Then, BugBase, which is an extended database of Greengenes, was utilized to predict the phenotypes of the gut flora 39.

### Untargeted metabolomics study

A Waters ACQUITY ultra-high-performance liquid chromatography system (Milford, MA) coupled with a Waters Q-TOF Micromass system (Manchester, UK) was used to perform sample analysis in both positive and negative ionization modes. Both polar ionic and lipid modes were used depending on the properties of the serum metabolites. The detailed procedures for sample preparation, HPLC-MS experiments, and generation of the peak-ion intensity matrix were described in our previous publication 16. The matrix was further filtered by removing peaks with a zero value in greater than 80% of samples. The threshold for the coefficient variation (CV) value in the quality control (QC) samples was set at 30% (as a standard in the assessment of data repeatability). Furthermore, the Wilcoxon rank-sum test was used to identify peaks that differed in abundance between the ACS-statins group and the ACS group. Then, SIMCA software (v14.1, Umetrics, Sweden) was utilized to perform orthogonal partial least squares discriminant analysis (OPLS-DA). Important peaks were selected on the basis of variable importance in the projection (VIP) value > 1 and *P* <0.05. Classification of selected peaks was achieved based on the molecular mass data (m/z). Online databases and criteria used for metabolite classification were described in detail in our previous publication 16.

### Multi-omics correlation analyses

Spearman correlations between important bacterial taxa, serum metabolites and clinical parameters were calculated in SPSS software. Correlations between features were visualized using the pheatmap package. A Sankey plot was utilized to present the multi-omics correlation as a whole with the R package networkD3.

### Metagenomic pathway prediction by PICRUSt2

PICRUSt2 was utilized to predict the metagenomic functional compositions 40. Pathways that were different in abundance between the ACS-statins and the ACS groups were obtained using Welch's t-test, and the Storey FDR was used to correct for multiple tests. STAMP software (v2.1.3) was utilized for statistical analyses and visualization of the identified pathways. The Spearman correlation between statin-associated pathways and important statin-associated ASVs was calculated with SPSS and visualized using the pheatmap package.

### *In-vitro* growth curves of statins-associated species and measurement of SCFA

Atorvastatin and rosuvastatin (pharmaceutical secondary standard, certified reference material, Sigma-Aldrich Co., St. Louis, MO, USA)) were utilized for the *in-vitro* experiments. *Parabacteroides merdae*, *Bifidobacterium longum subsp. longum* and *Anaerostipes hadrus* were obtained from the Institute of Microbiology, Chinese Academy of Sciences. The YCFA medium was freshly prepared according to the previous studies and autoclaved at 121 °C for 15 min 41. The three strains described above were cultured anaerobically at 37 °C in YCFA medium containing 0.2% DMSO, or atorvastatin (dissolved in 0.2% DMSO, final concentration: 10 μM and 100 μM) or rosuvastatin (dissolved in 0.2% DMSO, final concentration: 8 μM and 80 μM). The optical density (OD600) was measured for the bacterial growth curve. One milliliter of culture samples at logarithmic phase was mixed with one-milliliter of ethyl acetate, and the supernatants after extraction were used for SCFA analysis using a GCMS-QP2010 Ultra with an autosampler (Shimadzu Corporation, Japan) and an Rtx-wax capillary column (60 m×0.25 mm×0.25 μm, RESTEK, USA) as previous described 42. The Student's t test was used for the difference comparison and GraphPad Prism7 was used for data visualization.

## Results

### Characteristics of the study population

A total of 133 participants were enrolled in this study. All subjects were further divided into the control group (*N* = 30), ACS group (*N* = 67) and ACS-statins group (*N* = 36) according to diagnosis and medication. The characteristics and traditional cardiovascular risk factors for the participants are summarized in Table 1. In terms of severity of coronary artery lesions, we observed that the ACS-statins group exhibited a higher proportion of three-stenosed vessels (55.6%) than the ACS group (38.8%). However, the difference in the Gensini score, which is used to quantify atherosclerotic burden, was not significant 43. In addition, there was no significant difference in cardiac troponin I (cTnI) or high-sensitivity C-reactive protein (hsCRP), which are sensitive biomarkers for assessing myocardial damage and inflammation 44, 45, between the two disease groups. In terms of coexistent diseases, we observed that significantly more subjects from the ACS-statins group (83.3%) had hyperlipidemia (HLP) than those from the ACS group (46.3%) or control group (26.7%) (ACS-statins versus control: *P* < 0.001, ACS-statins versus ACS: *P* < 0.001, χ^2^ test). However, we discovered no significant difference in the blood lipid profiles (TC, TG, HDL-C and LDL-C) between the ACS-statins group and the ACS group. In addition, we observed that except for both disease groups exhibiting significantly higher proportions of subjects with type 2 diabetes mellitus (T2DM) than the control group (ACS-statins versus control: *P* < 0.001, ACS versus control: *P* = 0.01, χ^2^ test), patients from the ACS-statins group were even more likely to have T2DM than those from the ACS group (ACS-statins versus ACS: *P* = 0.048, χ^2^ test). In general, we believe that the difference in disease severity between the ACS-statins group and the ACS group was inconspicuous at baseline according to the results of cardiac catheterization and laboratory data, and a significantly larger proportion of subjects from the ACS-statins group had HLP and/or T2DM.

### Relatively better outcomes of patients from the ACS-statins group than those from the ACS group

Postdischarge follow-up was conducted. Among the 103 patients diagnosed with ACS, 68 (66.0%) patients were followed up and 35 (34.0%) patients were lost to follow-up due to loss of contact or personal rejection in rare cases. Among the 68 patients followed up, 42 patients were from the ACS group and 26 patients were from the ACS-statins group. The median follow-up time was 2.16 (IQR: 2.04-2.24) years. In the ACS group, composite endpoint events were observed in 10 subjects, including 1 cardiac death, 8 recrudescent, nonfatal ACS, and 1 nonfatal stroke, and there were 9 readmissions for cardiac causes. In the ACS-statins group, composite endpoint events were observed in 2 patients, including 2 recrudescent, nonfatal ACS, and there were 2 readmissions for cardiac causes. Binary logistic analyses demonstrated that statin therapy was associated with a decreased risk of ACS reoccurrence (odds ratio (OR) = 0.068, 95% CI: 0.006-0.730, *P* = 0.026), a decreased risk of readmission due to cardiac causes (OR = 0.118, 95% CI: 0.015-0.940, *P* = 0.044) and a decreased risk of composite endpoints (OR = 0.103, 95% CI: 0.013-0.799, *P* = 0.030) after adjusting for confounding factors, including the type of ACS (MI versus UA), male sex, age, history of old myocardial infarction (OMI), HLP, T2DM, hypertension (HTN), use of oral antidiabetic drugs (OAD) and use of antihypertensive drugs (Figure S1A-C, Table S1). In summary, patients who had accepted long-term and standard statin therapies tended to have a better prognosis in our study.

### Overview of the gut microbiome in different groups

In the present microbiome investigation, a total of 2,830,519 high-quality 16S rRNA reads were obtained, with a median read count of 20,597 (range: 10,048 to 40,210) per sample. A total of 829 ASVs were obtained after denoising. The rarefaction curves (Figure S2A) of all samples supported the adequacy of the sequencing depth. In terms of alpha diversity, we observed no significant differences in the Chao1 index (Figure S2B) or Shannon's index (Figure S2C) between the three groups. To assess the overall structure of the gut microbiota, a score plot of PCoA based on the weighted UniFrac distances (Figure S2D) was constructed. The results revealed a separation of the gut microbiota structure of the control group and ACS group (*P* = 0.020, Adonis test). However, no significant difference in beta diversity was observed between the ACS-statins group and the ACS or control group. Our results showed that although the overall gut microbiota composition of the ACS group differed from that of the control group, the ACS-statins group exhibited no significant difference in the overall structure of gut microbiota from either the ACS group or the control group. Additionally, the effect of dietary intake on the gut microbiota was summarized in Table S2. The explanation rate of each dietary factor on the structure of the gut microbiota was not statistically significant, although subjects from each group presented different diets.

**Figure 1:**
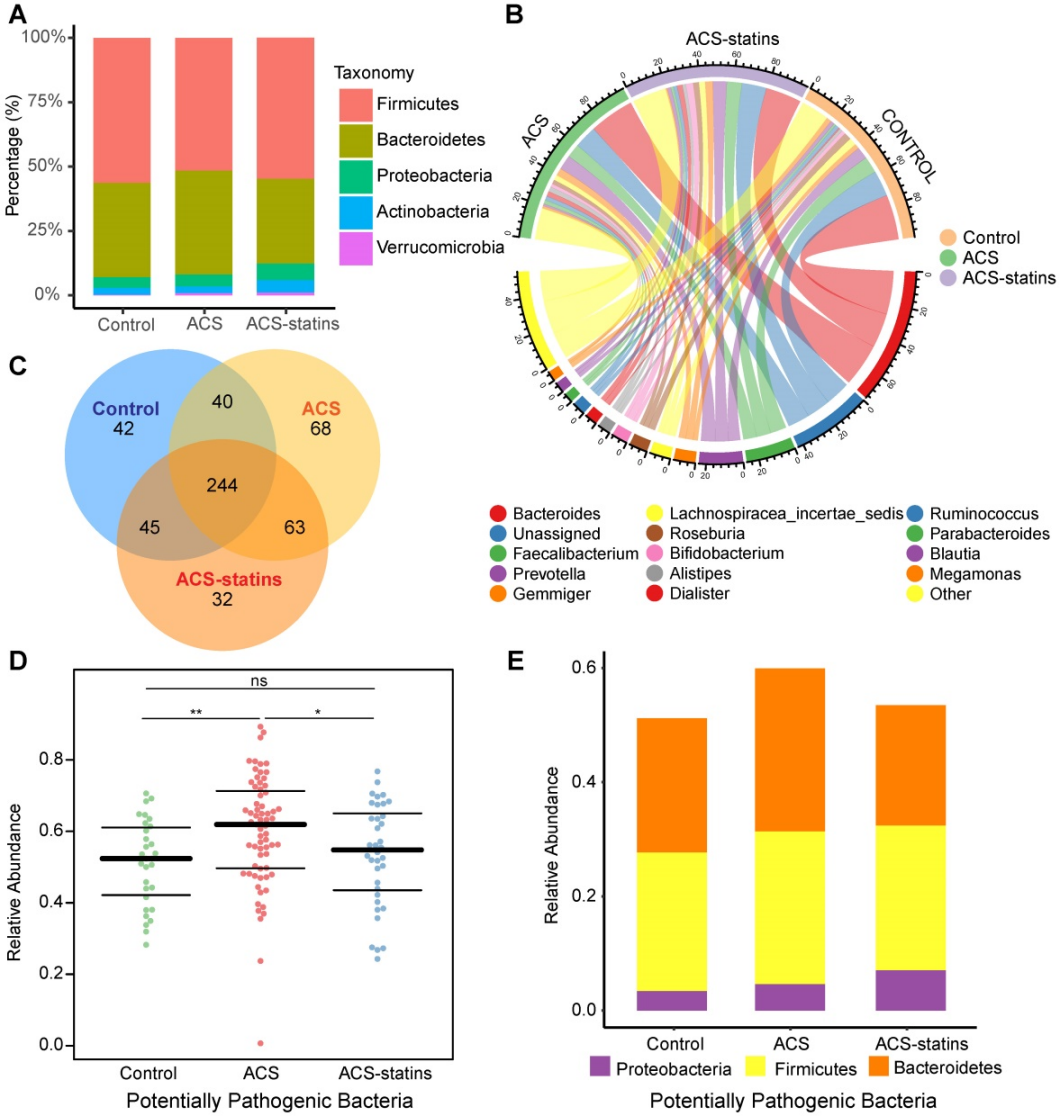
Overview of the gut microbiome in different groups. (A) Dominant phyla in each group. (B) Dominant genera and their contribution to each group. (C) A Venn diagram demonstrating the existence of ASVs in each group. (D) Relative abundance of potentially pathogenic bacteria predicted based on the BugBase database. *P < 0.05, **P < 0.01, ns: not significant, Mann-Whitney U test. (E) Distribution of potentially pathogenic bacteria at the phylum level in each group.

The relative proportion of dominant taxa at the phylum level was assessed and five phyla were identified in each group (Figure 1A). *Firmicutes* was the most dominant phylum, with a relative abundance of 56.4% in the control group, 51.5% in the ACS group and 54.8% in the ACS-statins group. The second most dominant phylum was *Bacteroidetes* (control: 36.6%, ACS: 40.4%, ACS-statins: 32.9%). Other observed phyla included *Proteobacteria*, *Actinobacteria* and *Verrucomicrobia*. Several of the most abundant genera at the genus level and their contribution to each group are shown in Figure 1B. *Bacteroides*, accounting for 27.0% of all samples, was the most predominant genus. A Venn diagram was constructed to examine the existence of ASVs with a relative abundance > 0.1% in each group (Figure 1C). Most ASVs (244 in all) were shared by all three groups. However, a total of 63 ASVs were specifically shared by two disease groups. Additionally, a total of 40 ASVs were shared by only the control group and the ACS group, and 45 ASVs were shared by only the control group and the ACS-statins group. In total, 68 ASVs were uniquely present in the ACS group, 42 ASVs in the control group and 32 ASVs in the ACS-statins group.

Phenotypes of the gut flora in each group were predicted based on the BugBase database. Interestingly, the sum of potentially pathogenic bacteria was particularly higher in the ACS group than in either the control group (*P* = 0.003, Mann-Whitney *U* test) or the ACS-statins group (*P* = 0.044, Mann-Whitney *U* test). The relative abundance and distribution of the potential pathogenic bacteria at the phylum level in each group are presented in Figure 1D-E.

### Alterations in the composition of fecal microbiota associated with statin use

**Figure 2:**
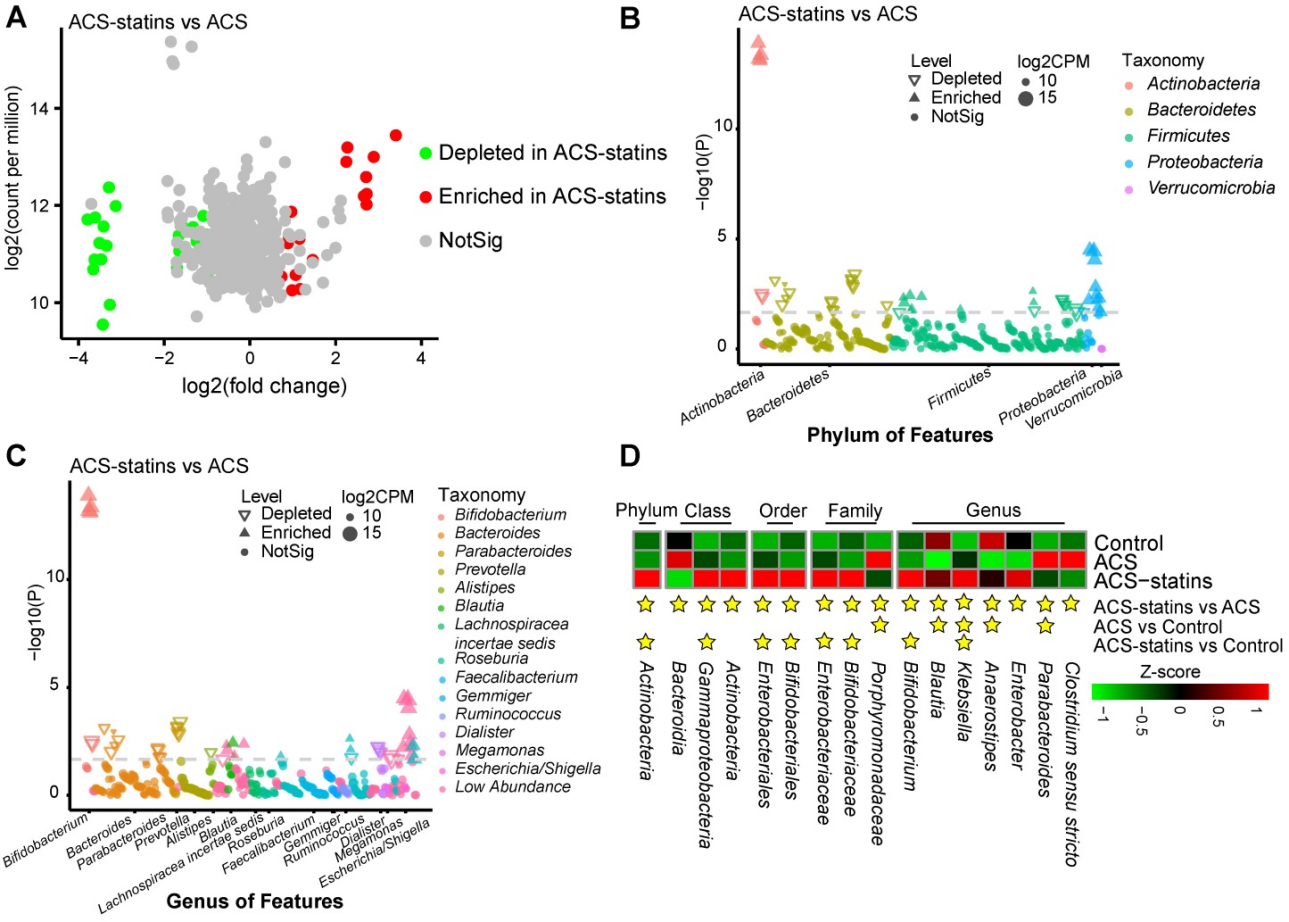
Alterations in the composition of fecal microbiota associated with statin treatment. (A) A volcano plot demonstrating differential ASVs between the ACS-statins group and the ACS group. (B) A Manhatten plot showing the distribution of differential ASVs at the phylum level. (C) A Manhatten plot showing the distribution of differential ASVs at the genus level. (D) A heatmap illustrating the relative abundance of statin-associated taxa across the three groups. A threshold of *P* < 0.05 and FDR < 0.2 calculated by edgeR was considered statistically significant.

To compare the differences in fecal microflora between groups, edgeR was utilized. A threshold of *P* < 0.05 and FDR < 0.2 was selected. We mainly focused on the significantly different taxa between the ACS group and the ACS-statins group, since we considered these taxa to be associated with statin use. As shown in the volcano plot (Figure 2A), a total of 51 ASVs exhibited significantly different abundances in the two disease groups, including 24 ASVs enriched in the ACS-statins group and 27 ASVs depleted in the ACS-statins group. Manhattan plots showed the contributions of differentially abundant ASVs at both the phylum level (Figure 2B) and genus level (Figure 2C). The relative abundance of these ASVs is shown in a heatmap (Figure S3).

The heatmap (Figure 2D) illustrated the relative abundance of statin-associated taxa across the three groups. At the phylum level, *Actinobacteria* was significantly more abundant in the ACS-statins group than in the ACS and control groups (ACS-statins versus ACS: FDR = 0.033, ACS-statins versus control: FDR = 0.010). At the genus level, we noted that *Blautia* (ACS-statins versus ACS: FDR = 0.032, control versus ACS: FDR = 0.008) and *Anaerostipes* (ACS-statins versus ACS: FDR = 0.005, control versus ACS: FDR < 0.001), both of which produce butyric acid 46-48, showed similar variation trends of abundance across the three groups: enriched in the ACS-statins group and control group, but depleted in the ACS group. Instead, *Parabacteroides* was significantly depleted in the ACS-statins group and control group but enriched in the ACS group (ACS versus ACS-statins: FDR = 0.144, ACS versus control: FDR = 0.014). Moreover, *Bifidobacterium*, which is a ubiquitous inhabitant of the gastrointestinal tract and generally considered a group of probiotics 49, was extremely more abundant in the ACS-statins group than in either the ACS group or the control group (ACS-statins versus ACS: FDR < 0.001, ACS-statins versus control: FDR = 0.009). In addition, although the difference was not statistically significant, *Bacteroides* tended to be less abundant in the ACS-statins group than in the ACS group (ACS-statins: 20.9%, ACS: 26.3%, *P* = 0.089), which aligned well with a previous report 50.

Furthermore, we correlated the statin-associated ASVs and genera to clinical phenotypes using the Spearman correlation method (Figure S4A-B, Figure S5A-B). Notably, two statin-enriched genera (*Blautia* and* Anaerostipes*) were negatively correlated with the Gensini score (*Blautia*: coefficient = -0.198, *P* = 0.023; *Anaerostipes*: coefficient = -0.178, *P* = 0.041). Moreover, *Anaerostipes* was negatively correlated with cTnI (coefficient = -0.248, *P* = 0.004). The above results suggest that *Blautia* and *Anaerostipes* are negatively correlated with the severity of ACS. However, we also found that the *Klebsiella* genus, which has been reported as an opportunistic pathogen and was enriched in the ACS-statins group (ACS-statins versus ACS: FDR < 0.001, ACS-statins versus control: FDR = 0.009, ACS versus control: FDR = 0.132), was positively correlated with T2DM (coefficient = 0.289, *P* < 0.001) 51. Since a significantly larger proportion of subjects from the ACS-statins group had T2DM than those from the other two groups and *Klebsiella* infection has been reported to be associated with T2DM and poor glycemic control 52, we speculated that the increased *Klebsiella* in the ACS-statins group was associated with the existence of T2DM.

### Changes in the serum metabolomic features associated with statins use

Since the gut microbiota usually interplays with host through complex metabolic pathways, we explored the serum metabolome through the untargeted LC-MS method. After QC and removal of peaks with low abundance, a total of 10,416 features in polar ionic mode and 3131 features in lipid mode were conserved. OPLS-DA models were applied to describe metabonomic distributions between the ACS-statins group and the ACS group. The OPLS-DA model was based on one predictive component and one orthogonal component. Pareto scaling was utilized for data normalization in OPLS-DA. The score scatter plots of the two modes are shown in Figure S6A-B. After thresholding metabolites with a VIP value > 1 and a Wilcoxon rank-sum *P* value < 0.05, a total of 47 metabolic features belonging to the polar ionic mode that significantly differed in abundance between the ACS-statins group and the ACS group were identified, including 43 features that could be classified based on online databases and 4 features that could not be classified (Table S3). The relative abundances of these metabolites across different groups were visualized as a heatmap (Figure S7A), and the VIP values of the top 20 metabolites are shown in a bar plot in Figure S7B.

**Figure 3:**
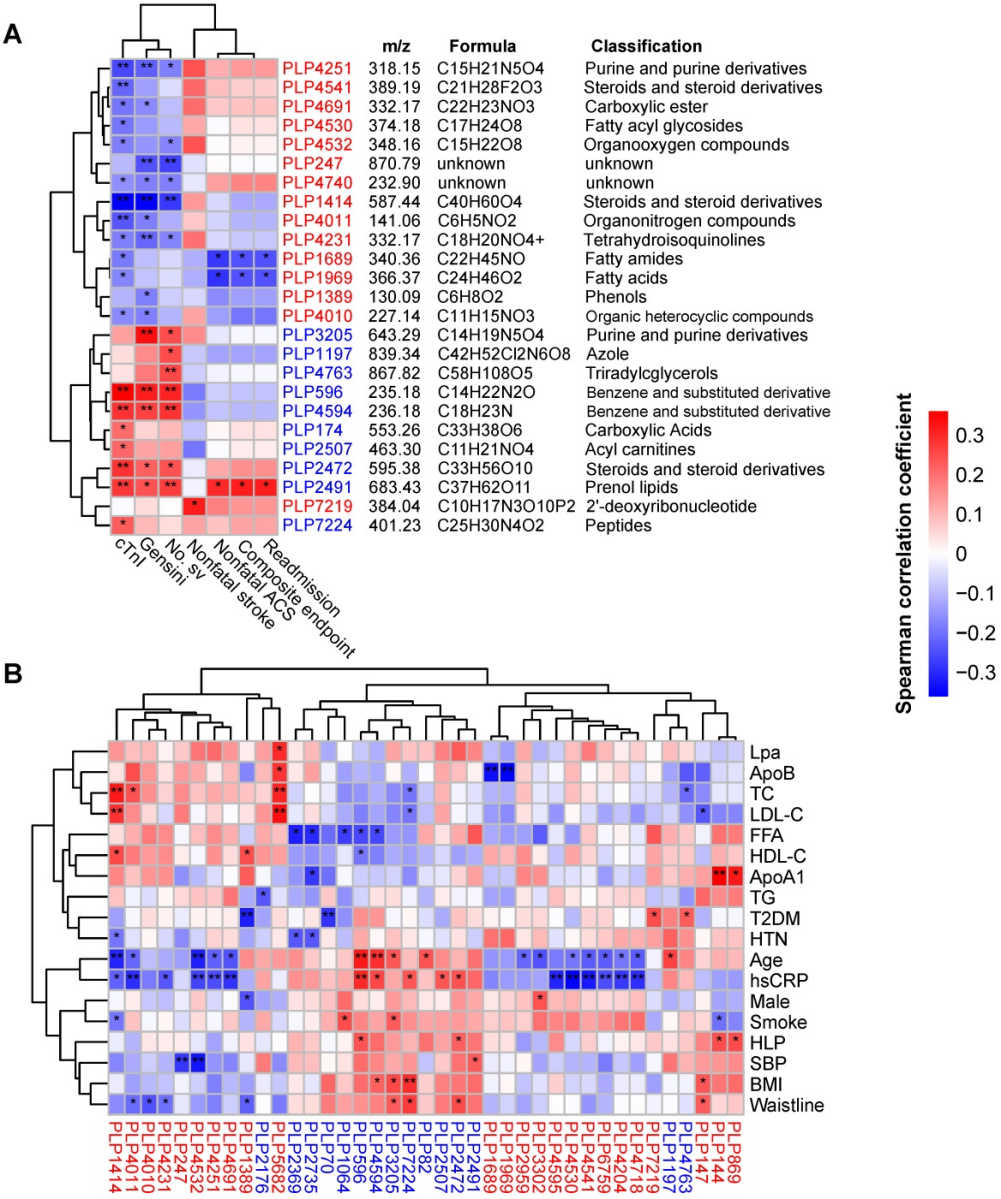
Identification of major serum metabolic features associated with the phenotypes of ACS. (A) Spearman correlations between statin-associated metabolic features and clinical outcomes as well as indicators of disease severity in patients with ACS. (B) Spearman correlations between statin-associated metabolitc features and major risk factors for ACS. The IDs of metabolic features are highlighted in red (statin-positive) and blue (statin-negative). *P < 0.05, **P < 0.01. Lpa: lipoprotein (a); ApoB: apolipoprotein B; FFA: free fatty acid; ApoA1: apolipoprotein A1.

Next, we correlated the differentially abundant metabolites with clinical phenotypes. As shown in Figure 3A, a total of 25 metabolites were correlated with indicators of disease severity (cTnI levels, Gensini score and number of stenosed vessels) and outcome events. Interestingly, 14 out of 15 statin-positive metabolites were negatively correlated with disease severity and were classified into fatty acyls, steroids and steroid derivatives, etc. However, all 10 statin-negative metabolites were positively correlated with disease severity and were classified into benzene and substituted derivatives, prenol lipids, acyl carnitines, etc. Notably, we observed that two statin-positive metabolic features (PLP1689 and PLP1969, both belonging to fatty acyls) were negatively correlated with adverse outcomes of patients (*P* < 0.05, Spearman correlation). In contrast, the statin-negative metabolic feature PLP2491 (cyclopassifloside II) was positively correlated with adverse outcomes of patients (*P* < 0.05, Spearman correlation). Correlations between the identified metabolites and main risk factors for ACS are shown in Figure 3B. We found that statin-positive metabolites (12 of 24, *P* < 0.05) exhibited a negative correlation with the levels of hsCRP, a sensitive biomarker used to evaluate the inflammatory status. Instead, statin-negative metabolites (5 of 15, *P* < 0.05) exhibited a positive correlation with hsCRP.

Taken together, these results suggest that statin therapy is associated with certain serum metabolite changes in ACS patients. Statins-positive metabolites tended to negatively correlate with disease severity and adverse outcome events, while statin-negative metabolites displayed opposite trends.

### Relationship between the gut microbiota and serum metabolites associated with statin therapy in ACS

**Figure 4:**
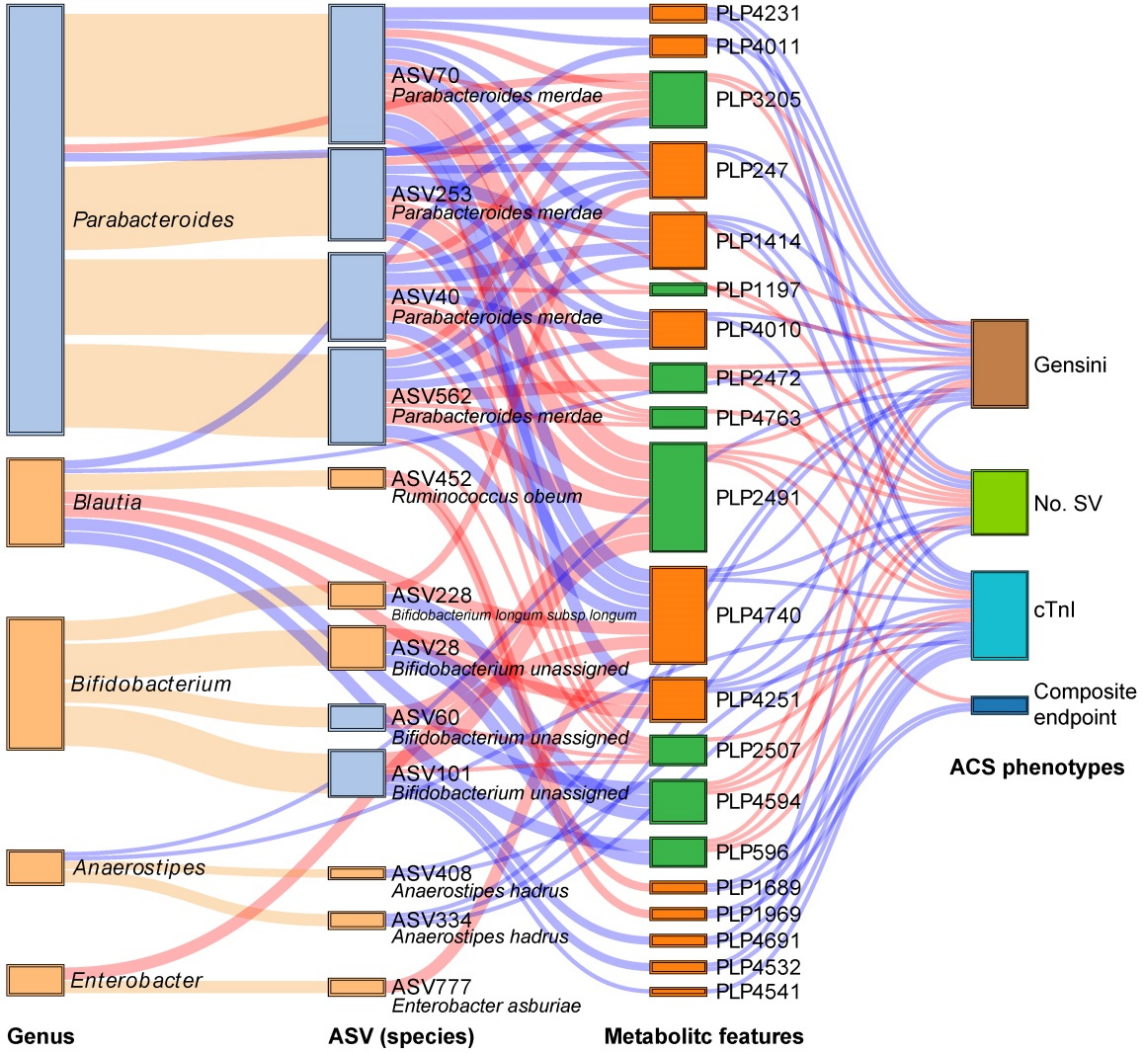
Interrelationship between statin-associated flora, serum metabolic features and major ACS phenotypes. A Sankey plot was utilized to examine the relationship between important statin-associated taxa and the outcomes of ACS patients as well as major parameters of disease severity either directly or by mediating key serum metabolic features. Red connections indicate positive correlations, and blue connections indicate negative correlations (Spearman correlation analysis, *P* < 0.05). Orange connections indicate the attribution of ASVs at the genus level. In the left two columns, light blue boxes indicate statin-negative genera or ASVs, and light orange boxes indicate statin-positive genera or ASVs. In the metabolic features column, green boxes indicate statin-negative metabolic features, and orange boxes indicate statin-positive metabolic features. A composite endpoint was defined as all-cause mortality and/or reoccurrence of ACS and/or readmission for cardiac causes. No. SV: number of stenosed vessels; cTnI: cardiac troponin I.

**Figure 5:**
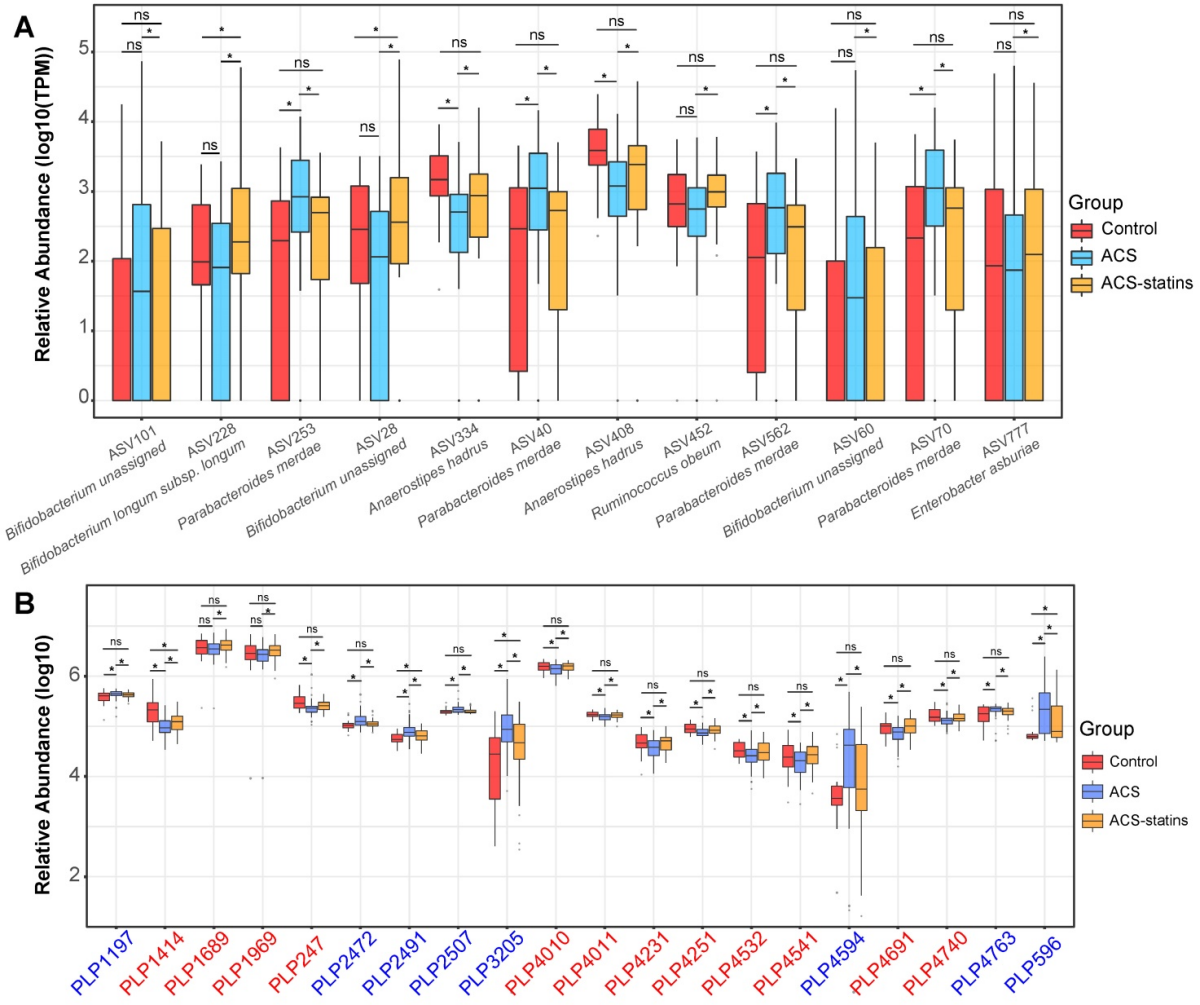
Relative abundance of key statin-associated ASVs and metabolic features in different groups. (A) Changes in important statin-associated ASVs among the three groups. ns: not significant, *P < 0.05, FDR < 0.2, edgeR test. (B) Changes in important statin-associated metabolic features among the three groups. ns: not significant, *P < 0.05, Wilcoxon rank-sum test. The IDs of metabolic features are highlighted in red (statin-positive) and blue (statin-negative).

We subsequently analyzed the Spearman correlation between the statin-associated microbial taxa and serum metabolites (Figure S8A-B). As demonstrated in Figure 4, a total of 12 statin-associated ASVs that contributed to 5 genera were significantly (*P* < 0.05) correlated with 20 metabolites, which were further correlated with indicators of disease severity and/or the composite endpoint. In addition, some taxa were directly correlated to clinical phenotypes, which were also visualized in the Sankey plot. At the ASV level, the statin-positive taxa that were directly or indirectly associated with the clinical phenotypes included *Ruminococcus obeum* (ASV452), *Bifidobacterium longum subsp. longum* (ASV228), *Anaerostipes hadrus* (ASV334 and ASV408) and *Enterobacter asburiae* (ASV777), while the statin-negative taxa associated with clinical phenotypes referred to *Parabacteroides merdae* (ASV40, ASV70, ASV253, and ASV562). We observed that the statin-positive taxa were negatively associated with poor clinical phenotypes either directly or by mediating metabolites. In contrast, statin-negative taxa were positively associated with poor clinical phenotypes either directly or by mediating metabolites. In general, metabolites that were positively correlated with statin-positive taxa were classified into fatty acids, fatty amides, etc. Moreover, metabolites that were positively correlated with statin-negative taxa were classified into benzene and substituted derivatives, prenol lipids, etc. Notably, ASV452 (*Ruminococcus obeum*) was negatively associated with the composite endpoint through the mediation of PLP1689 (docosanamide) and PLP1969 (nervonic acid), while *Parabacteroides merdae* was positively associated with the composite endpoint through the mediation of PLP2491 (cyclopassifloside II). The relative abundances of the identified taxa and metabolites that were associated with clinical phenotypes across different groups are shown in Figure 5. The results of the multi-omics analysis are summarized in Table S4. We also performed subgroup analyses of the above ASVs and metabolites. As shown in Figure S9A-C and Figure S10A-C, the variation of identified statin-associated ASVs and metabolites across the control, non-statin and statin group was basically consistent after we divided ACS patients into the UA, NSTEMI and STEMI subgroups.

### Prediction of metagenomic functional changes associated with statins

**Figure 6:**
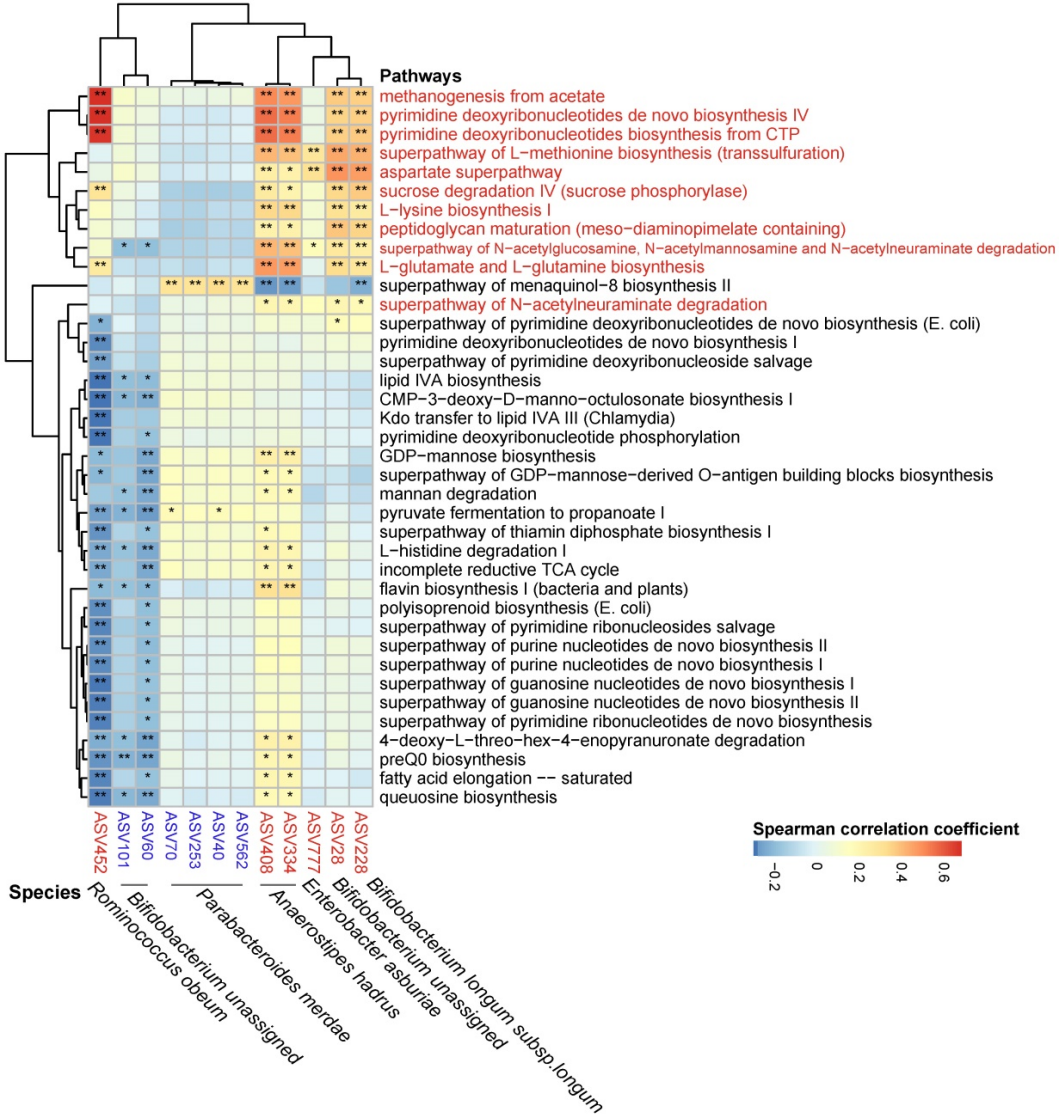
Spearman correlations between statin-associated ASVs and pathways. Spearman correlations were calculated between pathways predited by PICRUSt2 that differed between the ACS-statins and ACS groups and statin-associated ASVs. Rows: statin-positive pathways are highlighted in red, and statin-negative pathways are in black. Columns: statin-positive ASVs are highlighted in red, and statin-negative ASVs are highlighted in blue. *P < 0.05, **P < 0.01.

The metagenomic pathways were predicted using the PICRUSt2 tool based on the MetaCyc database 53. A total of 38 pathways were found to differ in abundance between the ACS group and the ACS-statins group (*P* < 0.05, FDR < 0.2, Figure S11, Table S5) (11 pathways enriched in the ACS-statins group and 27 pathways depleted in the ACS-statins group). Furthermore, these statin-associated pathways were correlated with important statin-associated ASVs (Figure 6). We observed that the methanogenesis pathway from acetate was positively correlated with statin-enriched species, especially *Ruminococcus obeum* (coefficient = 0.682, *P* < 0.001). Other pathways that were positively correlated with statins-enriched ASVs mainly included pyrimidine-associated pathways (e.g., pyrimidine deoxyribonucleotides de novo biosynthesis IV and pyrimidine deoxyribonucleotides biosynthesis from CTP), and amino acid-associated pathways (e.g., aspartate superpathway, L-lysine biosynthesis I, L-glutamate and L-glutamine biosynthesis, etc.). In addition, the statin-negative species *Parabacteroides merdae* was positively correlated with the menaquinal-8 biosynthesis II superpathway.

### Effects of statins on Parabacteroides merdae, Bifidobacterium longum subsp.longum and Anaerostipes hadrus *in vitro*

To validate the effects of statins on the growth of the identified species and bacterial production of metabolites, we performed *in-vitro* experiments. Growth curves of *Parabacteroides merdae*, *Anaerostipes hadrus* and *Bifidobacterium longum subsp. longum* under different concentrations of statins were analyzed. As shown in Figure S12A-B, the *in-vitro* growth of *Parabacteroides merdae* tended to be suppressed by statins. The inhibitory effect was especially evident with 100μM atorvastatin (*P* < 0.05, Student's t test, N = 3). In addition, statins significantly promoted the growth of *Anaerostipes hadrus* and *Bifidobacterium longum subsp. longum* in the logarithmic phase (Figure S12C-F, *P* < 0.05, Student's t test, N = 4). Moreover, Figure S12G-H shows that *Anaerostipes hadrus* and* Bifidobacterium longum subsp. longum* produced more butyrate in the presence of atorvastatin (*P* < 0.05, Student's t test, N = 3).

## Discussion

As the first-line agent for both primary and secondary prevention of CAD, statins exhibit prognostic benefits by lowering the incidence of cardiovascular events 54-56. Moreover, statins exhibit protective effects, even when they “fail” to prevent cardiovascular events, as in the case of ACS. In terms of pathological changes, statin therapy is associated with a reduced prevalence of ruptured plaques in patients with ACS 9. In addition, prior chronic treatment with statins is associated with fewer in-hospital complications and favorable outcomes at follow-up 10, 11, 57. In the present study, we also focused on the protective effects of statins in patients with ACS. We observed that patients who had received prior chronic treatment with statins exhibited fewer adverse outcomes than patients who had not, and we aimed to further understand the protective effects of statins by exploring the statin-associated changes in the gut microbiome as well as the serum metabolome in patients with ACS.

From a macro perspective, although we observed no significant difference in gut bacterial richness between groups according to the alpha diversity analysis, the beta diversity analysis suggested that the microbial composition of the ACS group differed significantly from that of the control group. However, we observed no significant difference in beta diversity between the ACS-statins group and either the ACS group or the control group, which suggested that the gut microbiota in the ACS-statins group was at an intermediate state. Importantly, we used the BugBase database and predicted that the potentially pathogenic bacteria were enriched in the ACS group but comparatively depleted in the ACS-statins group. Therefore, we speculate that statins could be a driving force to modulate the gut microbiome of ACS patients towards the status of healthy people.

Furthermore, we focused on specific taxa changes associated with statin use. According to Vieira-Silva et al.'s large population-based study, the *Bacteroides 2* (Bact2) enterotype, which is characterized by a high proportion of *Bacteroides*, is associated with systemic inflammation but negatively associated with statin treatment 50. In the present study, although the difference was not statistically significant, we still observed a downtrend of *Bacteroides* in the ACS-statins group compared to the ACS group, consistent with previous research. Notably, we observed that *Parabacteroides* exhibited characteristic disease-positive but statin-negative changes in the current study. Moreover, we specified the subordinate statin-negative ASVs into *Parabacteroides merdae* at the species level. Furthermore, our *in-vitro* experiments also supported the inhibitory effect of statins on the growth of *Parabacteroides merdae*. *Parabacteroides merdae* is a Gram-negative and obligately anaerobic bacterium that was reported to be enriched in the hypertensive gut microbiome 58, 59. In the present study, *Parabacteroides merdae* was positively associated with disease severity indicators through the mediation of PLP4594 (tolpropamine) and PLP596 (lidocaine), which are both drug-related benzenoids. Additionally, *Parabacteroides merdae* was positively associated with outcome events through the mediation of PLP2491 (cyclopassifloside II). Cyclopassifloside II (HMDB0038389), which belongs to the class of prenol lipids, is a glycosylated derivative of triterpene sapogenins. It can be derived from either food or endogenous synthesis. Statins can inhibit the synthesis of mevalonic acid, which is the precursor of prenol lipids 60, 61. Moreover, it has been reported that statins might exert cardioprotective effects by blocking the synthesis of isoprenoid intermediates 60. Therefore, we speculated that the decrease of serum cyclopassifloside II may be a result of statins. Although the relationship between cyclopassifloside II and gut bacteria remains ambiguous, we noticed that *Parabacteroides merdae* was positively correlated with the menaquinal-8 biosynthesis II pathway, which is an isoprenoid related pathway. Based on the above analyses, we believe further studies are needed to explore the causal relationship between *Parabacteroides merdae*, prenol lipid metabolism and ACS progression.

We also identified some statin-positive genera (*Bifidobacterium*, *Anaerostipes*, and *Blautia*) that were associated with protective effects. In the present study, *Bifidobacterium* was significantly increased in ACS patients who had received prior statin therapy, but we also observed that some ASVs of *Bifidobacterium* were statin-negative and associated with unfavorable clinical phenotypes, suggesting that *Bifidobacterium* functioned in a species/strain-dependent manner. *Bifidobacterium* is one of the main genera of commensal bacteria present in the human digestive tract, and some strains are considered probiotic microorganisms 62. *Bifidobacterium longum subsp. longum*, which was found to be statin-positive in our study, represents one of the most prevalent bifidobacterial species 63. It was reported to be a potential supplement in antiobesity treatment. According to Wu et al., the oral administration of *Bifidobacterium longum subsp. longum* reduced serum lipid profiles in mice fed with a high-fat diet 64. In the present study, we found no correlation between ASV228 (*Bifidobacterium longum subsp. longum*) and TC or LDL-C. However, we found that *Bifidobacterium longum subsp. longum* was negatively correlated with TG and BMI. Whether *Bifidobacterium longum subsp. longum* contributes to host lipid modulation synergistically with statins remains to be further explored.

Both *Anaerostipes* and *Blautia* are SCFA-producing genera. In our study, *Anaerostipes hadrus* was enriched in both the control and ACS-statins groups but depleted in the ACS group. In addition, it was negatively correlated with indicators of disease severity. *Anaerostipes hadrus* is a Gram-positive bacterium known for its ability to produce butyric acid 48. Zeevi et al.'s research on gut microbiota genomic structural variants (SVs) uncovered that a region in *Anaerostipes hadrus* that encodes a composite inositol catabolism-butyrate biosynthesis pathway is associated with low host metabolic risk assessed by BMI, waist-to-hip ratio, etc. The authors hypothesized that by processing this SV, bacteria demonstrated increased symbiosis with the host 65. In the current study, we found a significant negative correlation between *Anaerostipes hadrus* (ASV334 and ASV408) and the waistline, which is consistent with the previous report. Moreover, the statin-positive genus *Blautia* is also reported to have a strong negative association with metabolic risk. A previous 16S rRNA sequencing study demonstrated that the depletion of *Blautia* was pronounced in the gut microbiota of obese children, especially in those with insulin resistance 66. The relative abundance of *Blautia* was inversely associated with the visceral fat area, which is a risk factor strongly associated with cardiovascular disease and overall mortality 46. Correspondingly, a randomized controlled-feeding trial revealed that a lower-fat diet was associated with an increased abundance of *Blautia* 67. In our study, we found that *Ruminococcus obeum*, which was reclassified as *Blautia obeum comb. nov* in 2015 68, was negatively related to the composite endpoint of ACS patients through the mediation of PLP1969 (nervonic acid) and PLP1689 (docosanamide). Nervonic acid (HMDB0002368) is a long chain unsaturated fatty acid that is enriched in sphingomyelin. Docosanamide (HMDB0000583) is a carboxylic acid amide derivative of fatty acids. The biosynthesis of both metabolites remains to be illustrated. Except for endogenous synthesis and food, it has been reported that bacterial production could be a source of nervonic acid 69. In terms of biological function, *Ruminococcus obeum* (ASV452) was found to be strongly correlated with the pathway of methanogenesis from acetate predicted by PICRUSt2. In addition, *Ruminococcus obeum* has been reported as a propionate producer 70.

Based on the above analyses, we speculated that statin therapy might increase the relative abundance of bacteria that participate in fatty acid metabolism, thus benefiting the ACS patients. Therefore, we performed *in-vitro* culture of specific statin-positive bacteria. We observed that the logarithmic growth of *Anaerostipes hadrus* and *Bifidobacterium longum subsp. longum* was promoted by atorvastatin and rosuvastatin. Moreover, the above two bacteria produced more butyrate in the presence of atorvastatin. Butyrate, together with acetate and propionate, consist of so-called SCFAs. SCFAs have been well accepted as a class of bacterial products that are beneficial for host health. By inhibiting histone deacetylases (HDACs) or regulating G-protein coupled receptors (mainly GPR41 and GPR43), SCFAs exhibit anti-inflammatory effects and help improve host insulin sensitivity 70.

The gut microbial ecosystem is well accepted as a necessary endocrine organ that is capable of producing a wide range of biologically active compounds that may be released into the peripheral circulation and interact with multiple organs of the host. Our multi-omics study provided a general view of statin-associated gut microbiota as well as serum metabolomic changes. The correlation analysis and functional prediction analysis provided insights into how statins may benefit host metabolic balance by modulating gut microbiota in addition to directly blocking the biosynthesis of cholesterol. Further *in-vitro* experiments validated the role of statins on growth as well as butyrate production of specific statin-associated species. However, as the human gut micro-ecology is rather complex, the *in-vitro* single-strain culture may not simulate the real gut micro-environment *in vivo*, and statins may regulate gut microbes through complex pathways rather than directly interacting with bacteria. There are other limitations in this study. Although our follow-up study revealed that chronic prior statin therapy was associated with an improved outcome in patients with ACS, we only assessed the microbial and metabolomic features at baseline instead of monitoring the dynamic changes, and many confounding factors, such as medications aside from statins, diet and lifestyle, may result in bias in the correlation analysis. Additionally, due to technological limitations, the identification of serum metabolites by untargeted LC-MS may not be precise enough, and targeted metabolomic studies are needed to accurately validate specific changes in metabolites. Furthermore, the data interpretation might be limited due to the relatively small sample size, and the conclusions need to be validated by larger studies. Nonetheless, our study supports a regulatory role of statins on the gut microbiota that may explain the protective effects of statins in ACS, though further validation and exploration via functional studies are required.

## Conclusion

As the first-line agent for primary and secondary prevention of CAD, statins exhibit cardiovascular protective effects in a heterogenic manner rather than simply by blocking the biosynthesis of cholesterol. Our follow-up study demonstrated that prior chronic statin therapy was associated with an improved outcome in ACS patients. Multi-omics study revealed that statins may benefit the host by modulating the gut microbiome of ACS patients towards a healthier status, i.e., reducing potentially pathogenic bacteria such as* Parabacteroides merdae* and increasing beneficial bacteria such as *Bifidobacterium longum subsp. longum*, *Anaerostipes hadrus* and *Ruminococcus obeum*, thus reducing the metabolic risk of patients. Our findings provide new insights into the heterogenic roles of statins and multi-omics interactions in ACS, but further exploration by functional studies is required.

## Supplementary Material

Supplementary figures and methods.

Supplementary tables.

## Figures and Tables

**Table 1:** Characteristics of the study cohort

	Control (N=30)	ACS (N=67)	ACS-statins (N=36)	*P* value
Age*	54.87±7.65	62.03±10.95	62.33±9.52	0.003^bc^
Male*	12 (40.0)	48 (71.6)	29 (80.6)	<0.001^bc^
SBP*	117.80±9.56	131.64±18.94	125.75±14.94	0.001^bc^
BMI*	24.35±2.82	26.47±3.23	26.52±3.09	0.005^bc^
**No. sv^§^**				
NA	NA	2 (3.0)	3 (8.3)	NA
1	NA	22 (32.8)	7 (19.4)	NA
2	NA	17 (25.4)	6 (16.7)	NA
3	NA	26 (38.8)	20 (55.6)	NA
**Type of ACS^§^**				
UA	NA	48 (71.6)	27 (75.0)	NA
NSTEMI	NA	12 (17.9)	3 (8.3)	NA
STEMI	NA	7 (10.5)	6 (16.7)	NA
Gensini^#^	NA	32.5 (18,55)	33 (22,50)	ns
**Medications**				
HTN drug^§^	4 (13.3)	39 (58.2)	24 (66.7)	<0.001^bc^
OAD^§^	1 (3.3)	17 (25.4)	16 (44.4)	0.001^abc^
Smoke history^§^	4 (13.3)	39 (58.2)	20 (55.6)	<0.001^bc^
Drink history^§^	3 (10.0)	35 (52.2)	19 (52.8)	<0.001^bc^
HTN^§^	6 (20.0)	44 (65.7)	23 (63.9)	<0.001^bc^
T2DM^§^	1 (3.3)	17 (25.4)	16 (44.4)	0.001^abc^
HLP^§^	8 (26.7)	31 (46.3)	30 (83.3)	<0.001^ac^
OMI^§^	0 (0.0)	13 (19.4)	2 (5.6)	0.009^b^
**Laboratory data**				
TC^*^	4.81±0.87	4.02±0.88	3.81±1.30	<0.001^bc^
TG^*^	1.54±0.95	1.64±0.70	1.65±1.01	ns
HDL-C^*^	1.22±0.35	0.97±0.23	0.92±0.15	<0.001^bc^
LDL-C^*^	2.82±0.75	2.35±0.76	2.14±1.04	0.005^bc^
hsCRP^†^	0.66 (0.37,1.21)	2.13 (0.87,4.11)	2.03 (0.64,3.43)	0.001^bc^
cTnI^†^	0.00 (0.00,0.00)	0.01 (0.00,0.05)	0.01 (0.00,0.04)	<0.001^bc^

*mean ± SD, ^§^n (%), #median (IQR). For the difference comparison of clinical characteristics among the three groups, one-way analysis of variance (ANOVA) was employed in cases of continuous normally distributed data. The Bonferroni test was applied for post hoc comparisons in cases of equal variance, and the Tamhane test was applied in cases of unequal variance. The Kruskal-Wallis H-test was applied for continuous data that were not normally distributed among the three groups, and the Mann-Whitney *U* test was applied for this kind of data between two groups. Categorical variables were compared by the χ^2^ test or Fisher's exact test. NA: not available. SBP: systolic blood pressure. BMI: body mass index. No.sv: number of stenosed vessels. HTN: hypertension. OAD: oral anti-diabetic drugs. T2DM: type 2 diabetes mellitus. HLP: hyperlipidemia. OMI: old myocardial infarction. TC: total cholesterol. TG: triacylglycerol. HDL-C: high-density lipoprotein cholesterol. LDL-C: low-density lipoprotein cholesterol. hsCRP: high sensitivity C-reactive protein. cTnI: cardiac troponin I. a: ACS-statins vs ACS. b: ACS versus Control. c: ACS-statins vs Control.
