# Supplementary material for: Multi-omics study reveals that statin therapy is associated with restoration of gut microbiota homeostasis and improvement in outcomes in patients with acute coronary syndrome
Source: Theranostics. 2021 Mar 31;11(12):5778–93. doi: 10.7150/thno.55946 (PMC8058718; doi:10.7150/thno.55946)
Supplement: Supplementary file 1 — Supplementary figures and methods. [file thnov11p5778s1.pdf]

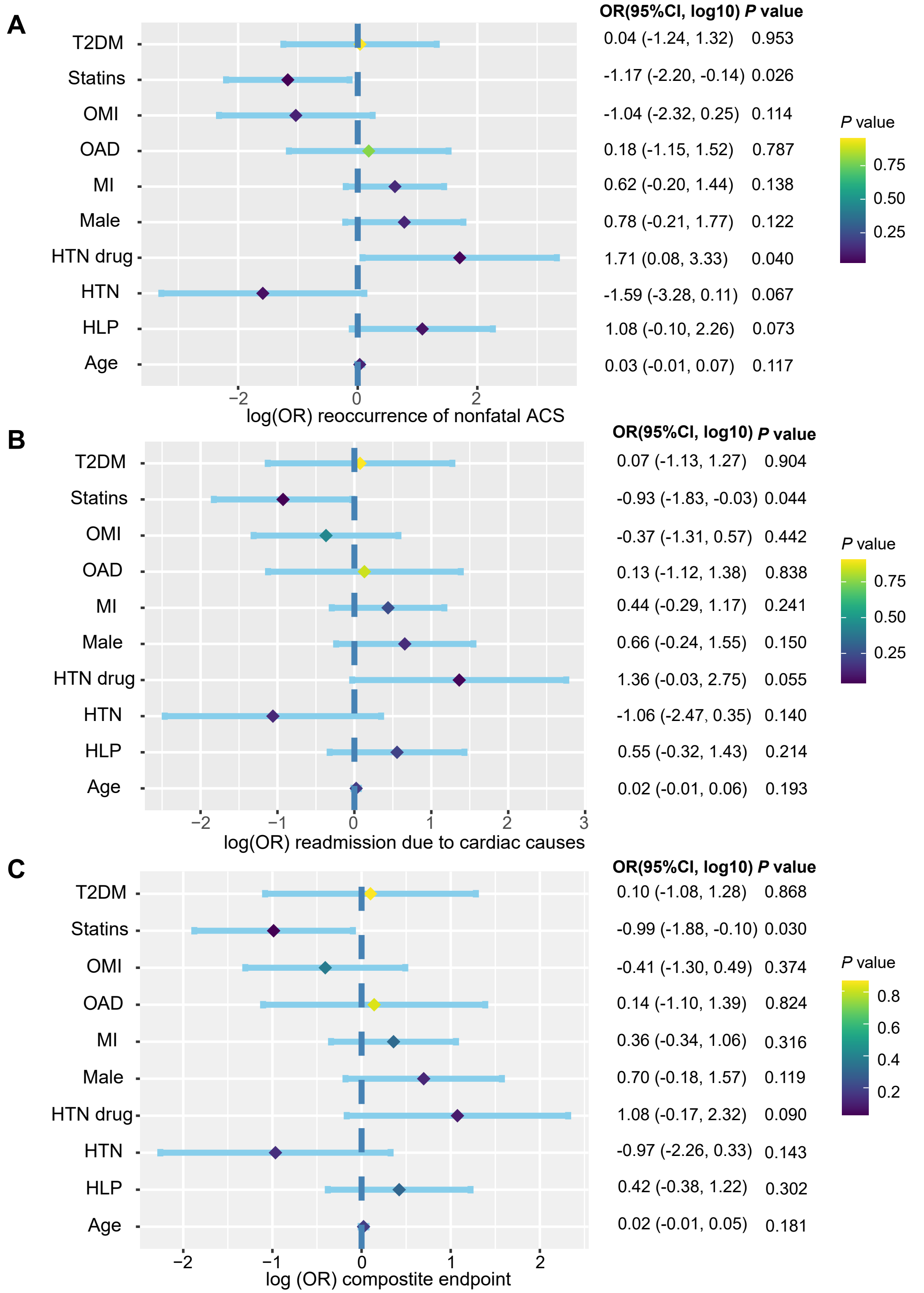

Figure S1. Forest plots demonstrating the results of binary logistic regression analysis. (**A**) Association between prior statin treatment and the reoccurrence of nonfatal ACS. (**B**) Association between prior statin treatment and readmission due to cardiac causes. (**C**) Association between prior statin treatment and the composite endpoint. Adjusted by T2DM, OMI, OADs, MI, male sex, HTN drugs, HTN, HLP and age.

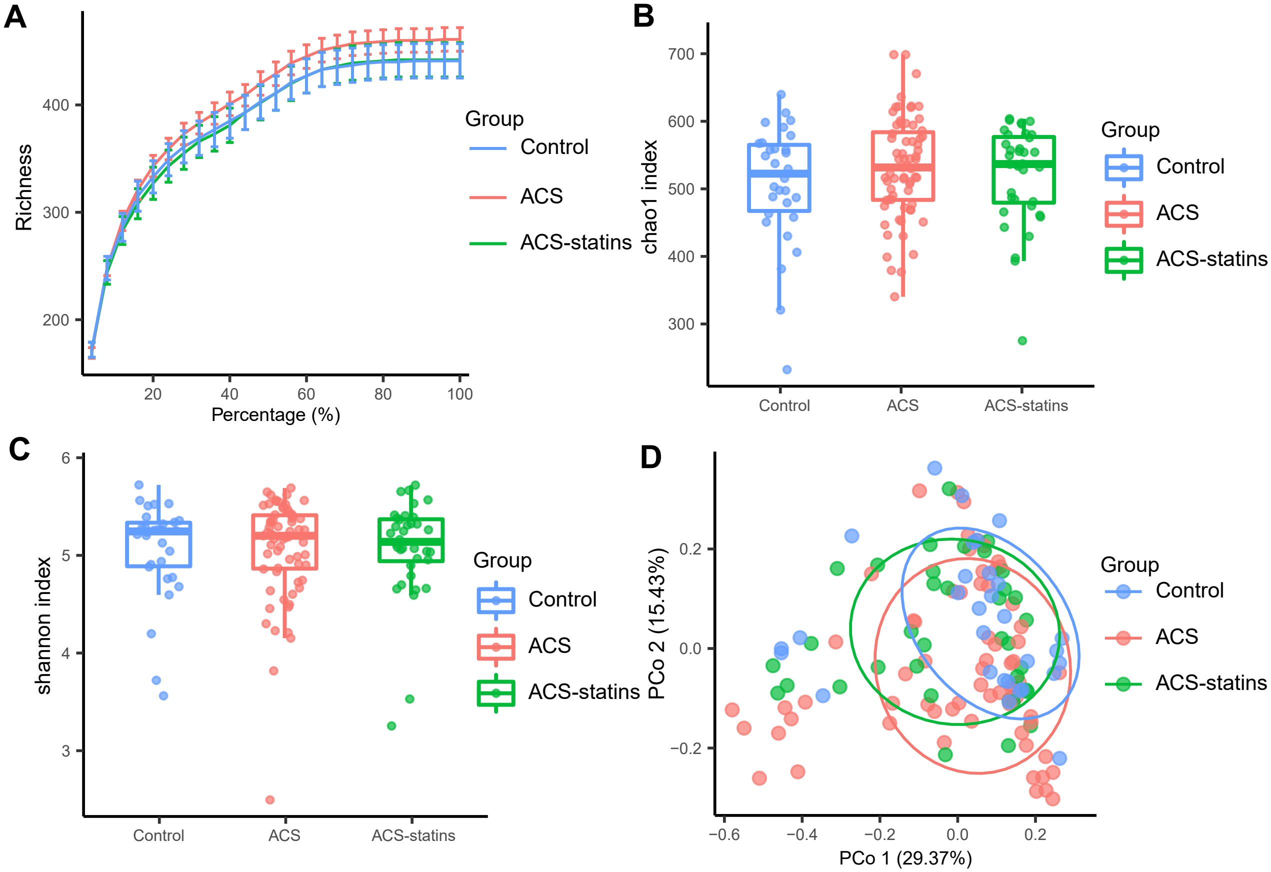

Figure S2. Gut microbiome diversity analysis. (**A**) Rarefaction curves. (**B**) Alpha diversity analyzed by the Chao1 index. (**C**) Alpha diversity analyzed by Shannon's index. (**D**) Beta diversity demonstrated by PCoA based on UniFrac distances (ACS versus control: $P = 0.020$, ACS-statins versus control: $P = 0.106$, ACS-statins versus ACS: $P = 0.241$, Adonis test).

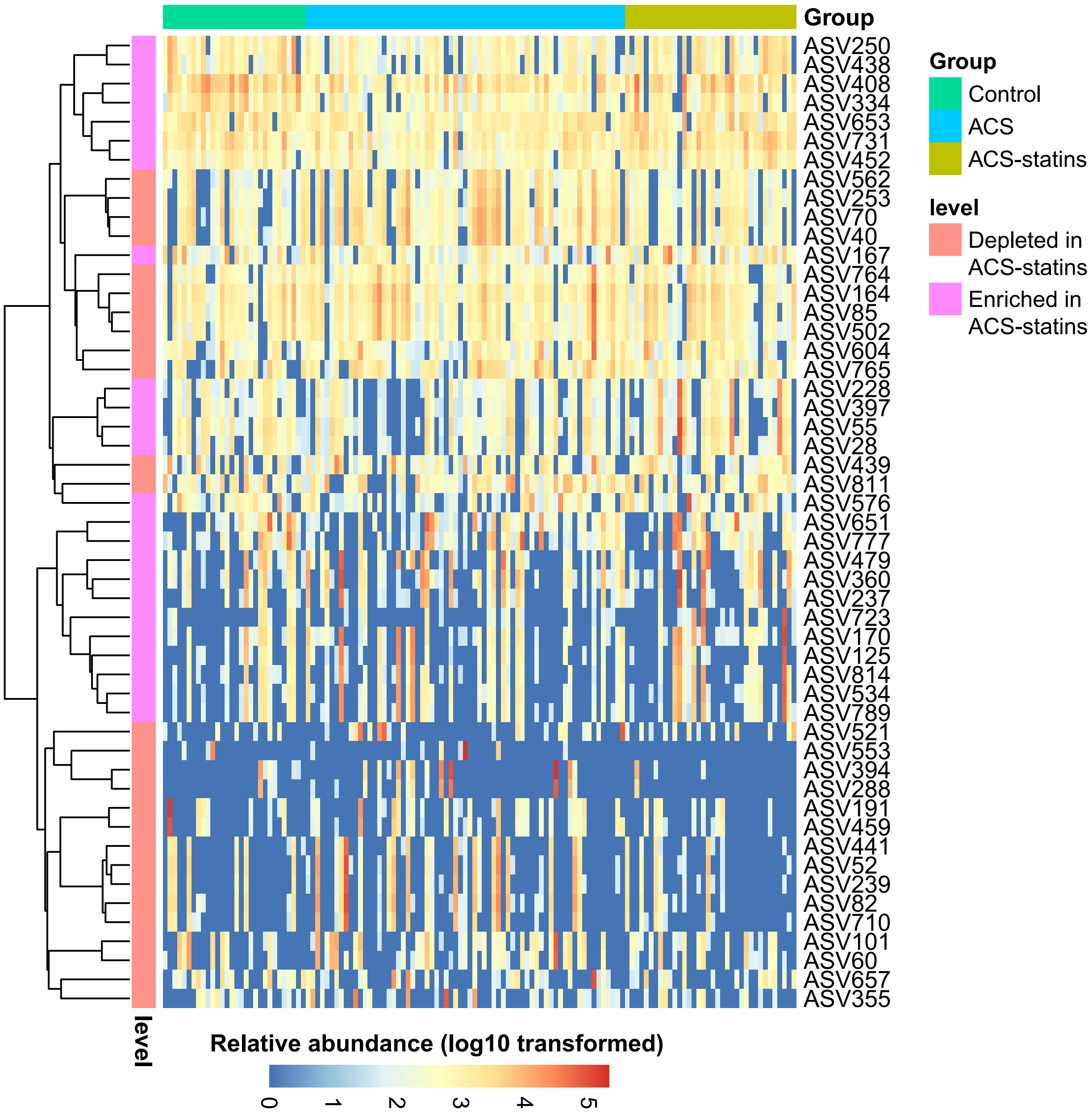

Figure S3. Relative abundance of differential ASVs between the ACS-statins group and the ACS group across samples.

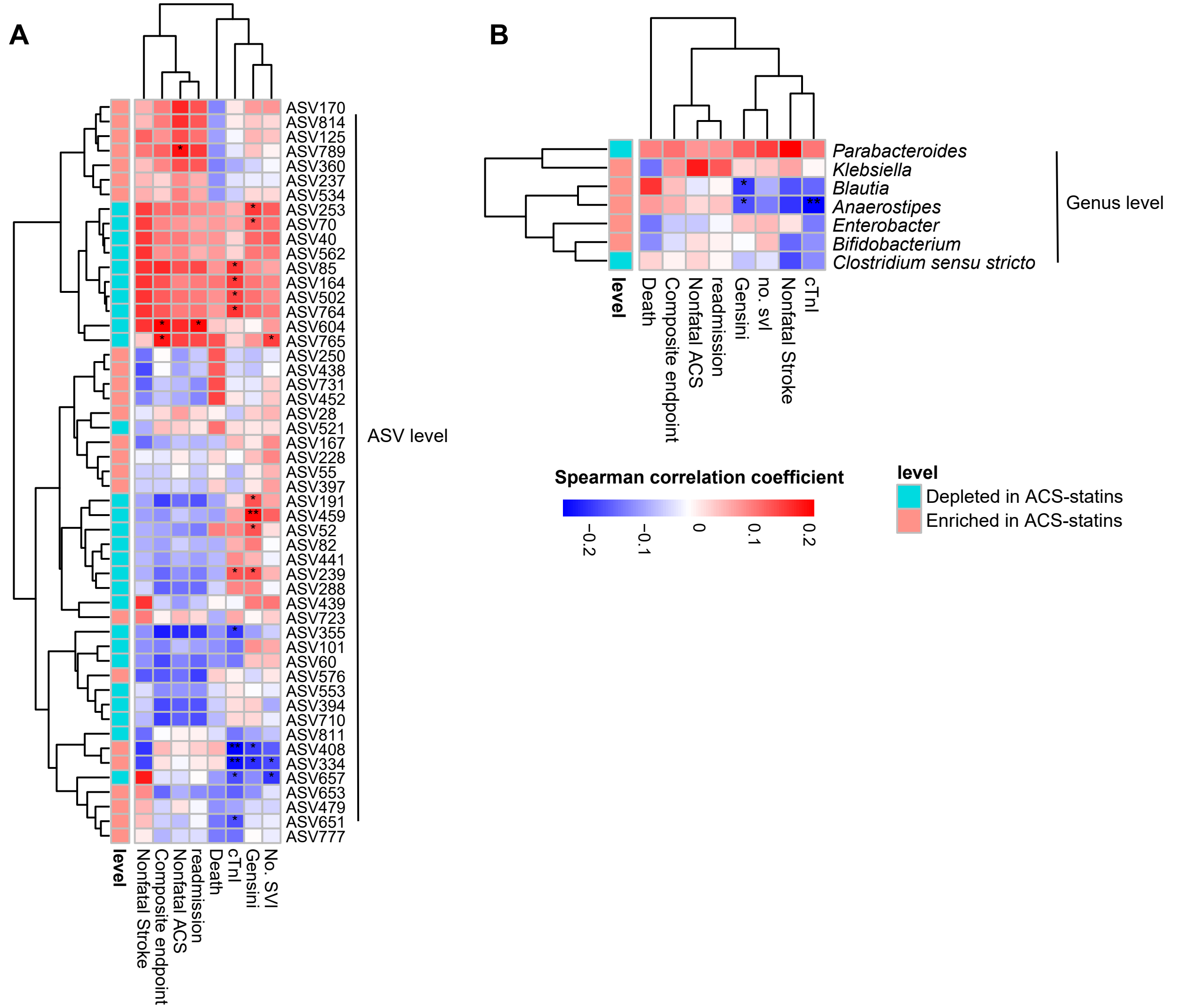

Figure S4. Spearman correlations between statin-associated gut flora and disease severity as well as adverse outcomes of ACS patients. (**A**) Correlations between statin-associated ASVs and phenotypes. (**B**) Correlations between statin-associated genera and phenotypes. * $P < 0.05$, ** $P < 0.01$.

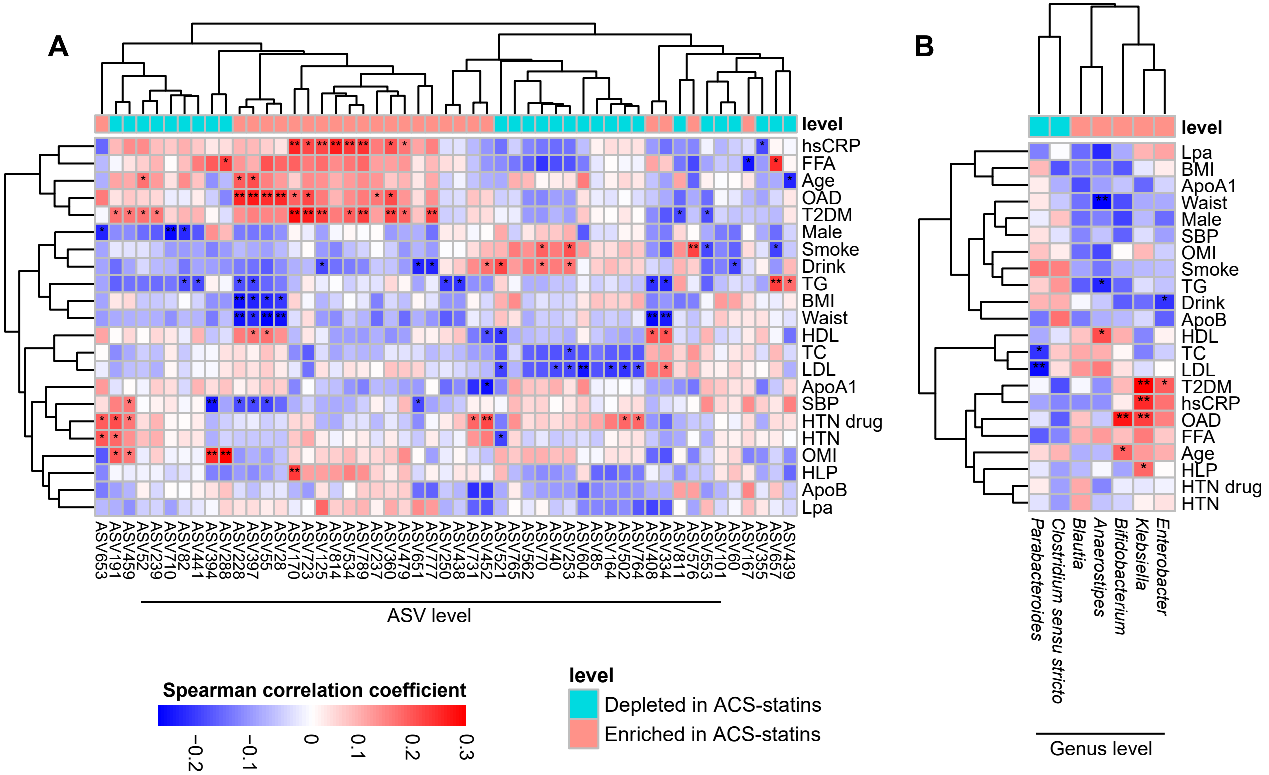

Figure S5. Spearman correlations between statin-associated gut flora and major risk factors for ACS. (**A**) Correlations between statin-associated ASVs and risk factors. (**B**) Correlations between statin-associated genera and risk factors. * $P < 0.05$, ** $P < 0.01$.

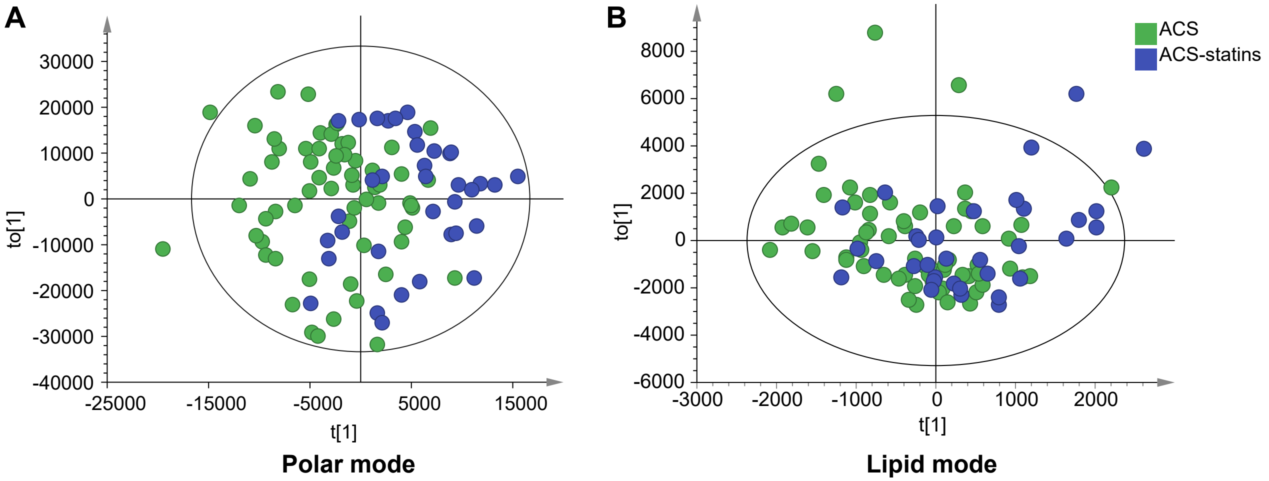

Figure S6. Orthogonal partial least squares discriminant analysis (OPLS-DA) score plots. (**A**) Score scatter plot under polar mode. (**B**) Score scatter plot under lipid mode. A comparison between the ACS and ACS-statins groups was performed.

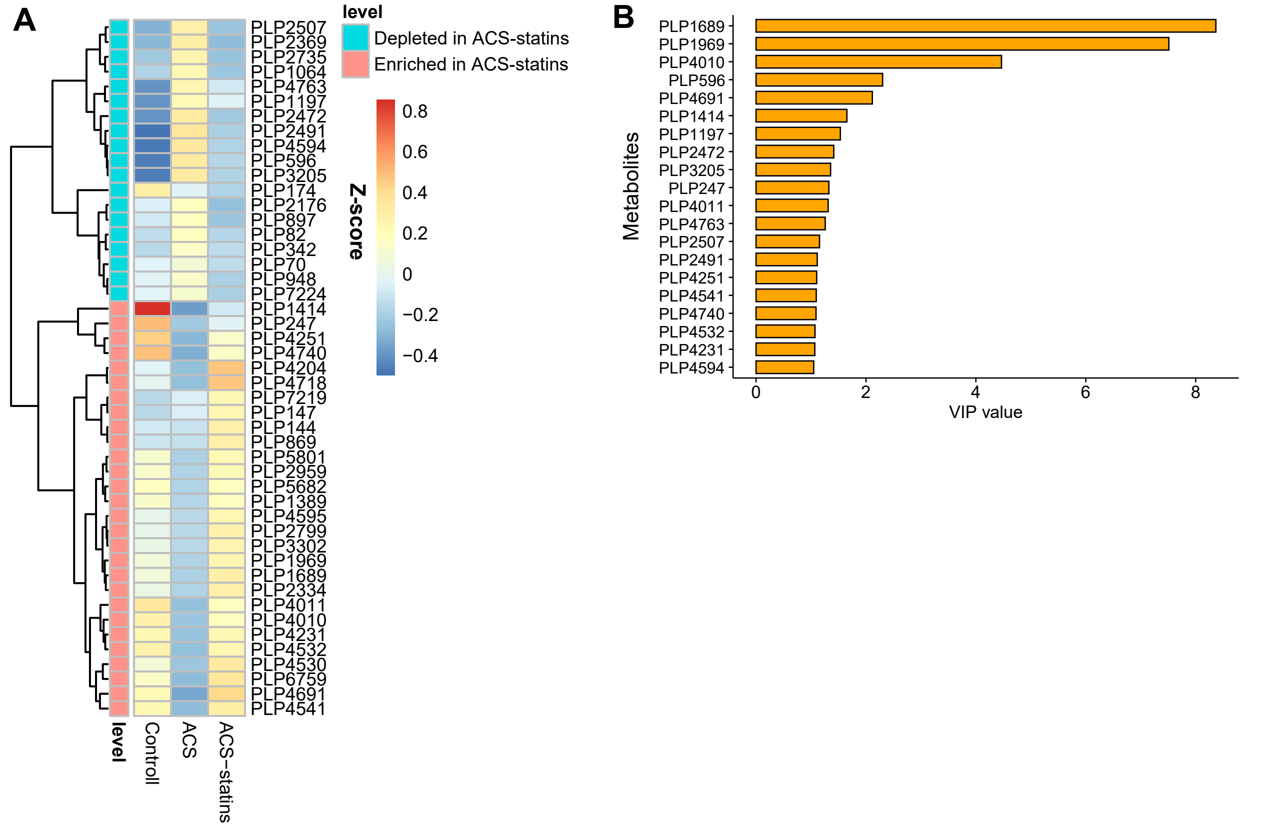

Figure S7. Relative abundances and VIP values of statin-associated metabolic features. (**A**) Heatmap demonstrating the relative abundances of statin-associated metabolites across the three groups. (**B**) VIP values of the top 20 statin-associated metabolites.

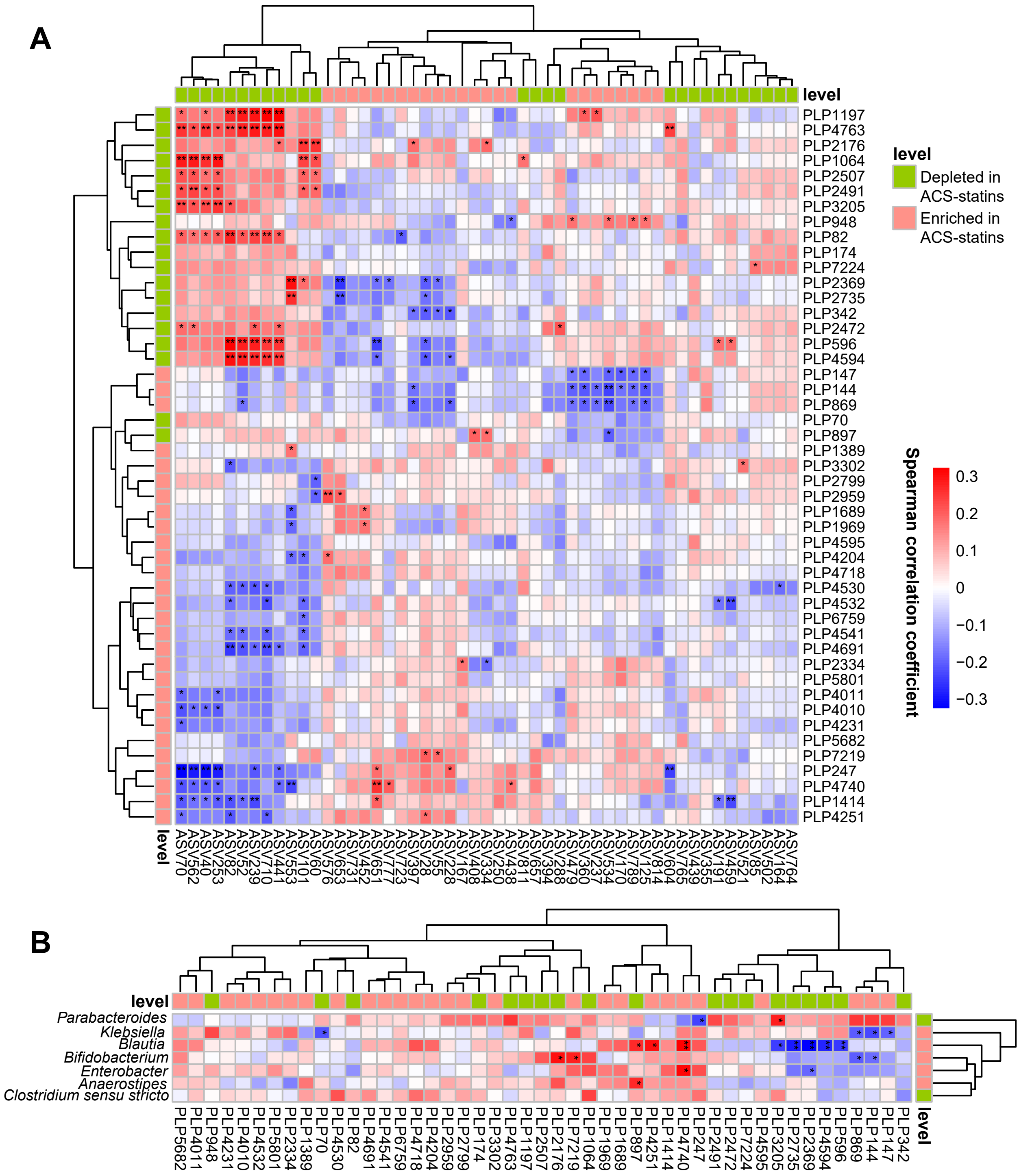

Figure S8. Spearman correlations between statin-associated bacteria and statin-associated serum metabolites. (**A**) Correlations between statin-associated ASVs and metabolites. (**B**) Correlations between statin-associated genera and metabolites.

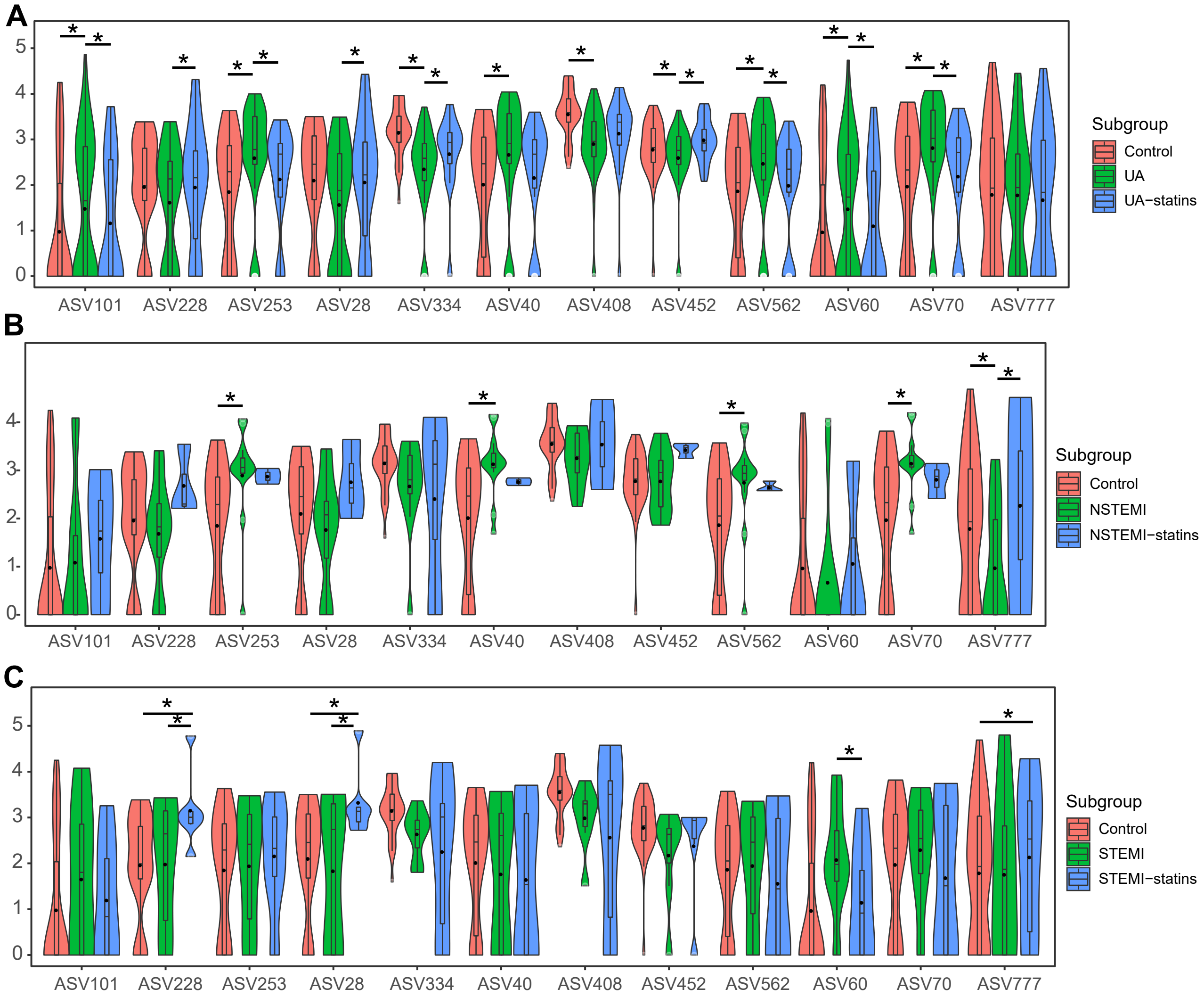

Figure S9. Comparison of statin-associated ASVs between subgroups. (**A**) Comparison of identified statin-associated ASVs between Control, UA and UA-statins groups. (**B**) Comparison of identified statin-associated ASVs between Control, NSTEMI and NSTEMI-statins groups. (**C**) Comparison of identified statin-associated ASVs between Control, STEMI and STEMI-statins groups. * $P < 0.05$, Wilcoxon rank-sum test.

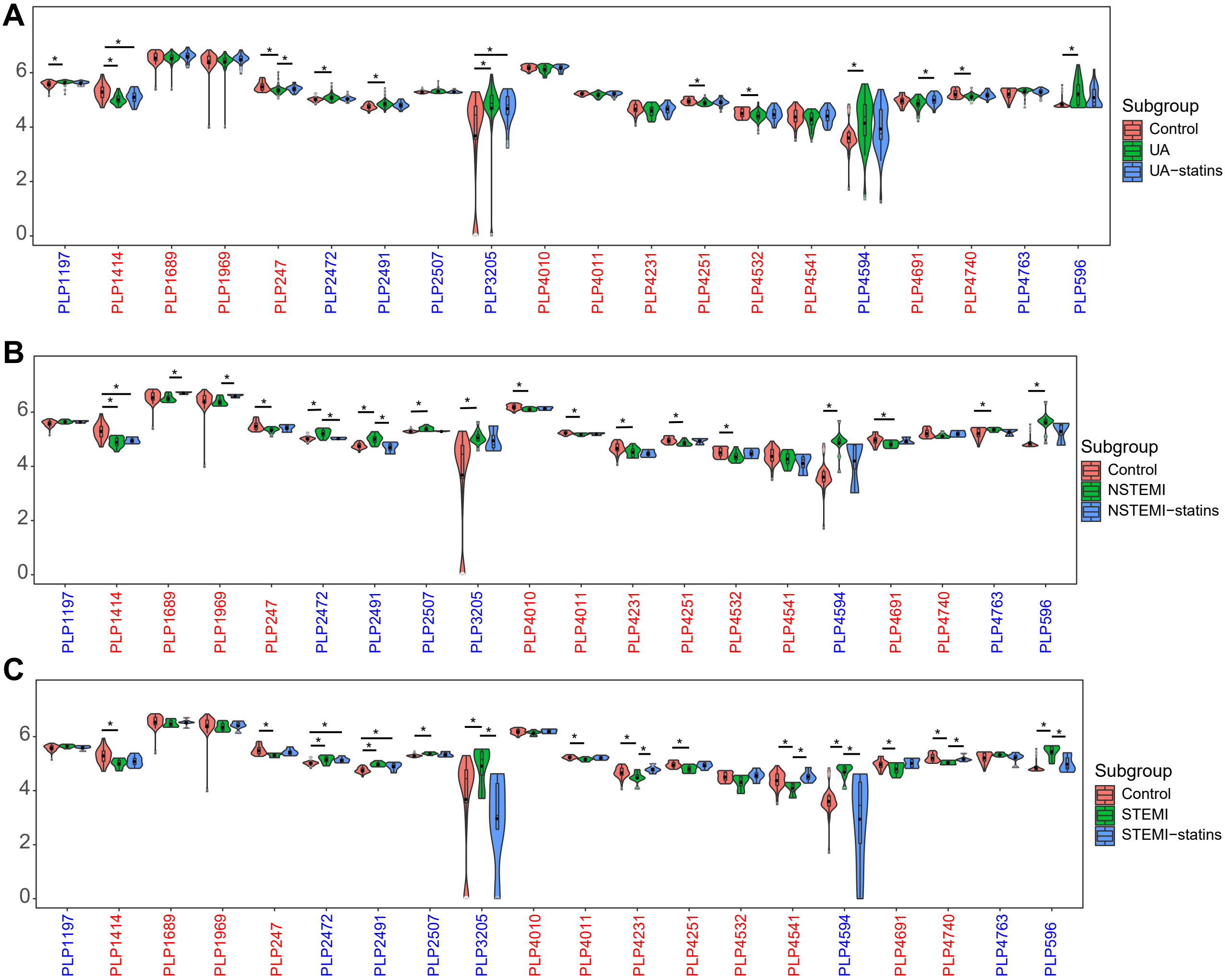

Figure S10. Comparison of statin-associated metabolic features between subgroups. (**A**) Comparison of identified statin-associated metabolites between Control, UA and UA-statins groups. (**B**) Comparison of identified statin-associated metabolites between Control, NSTEMI and NSTEMI-statins groups. (**C**) Comparison of identified statin-associated metabolites between Control, STEMI and STEMI-statins groups. * $P < 0.05$, Wilcoxon rank-sum test. The IDs of metabolic features are highlighted in red (statin-positive) and blue (statin-negative).

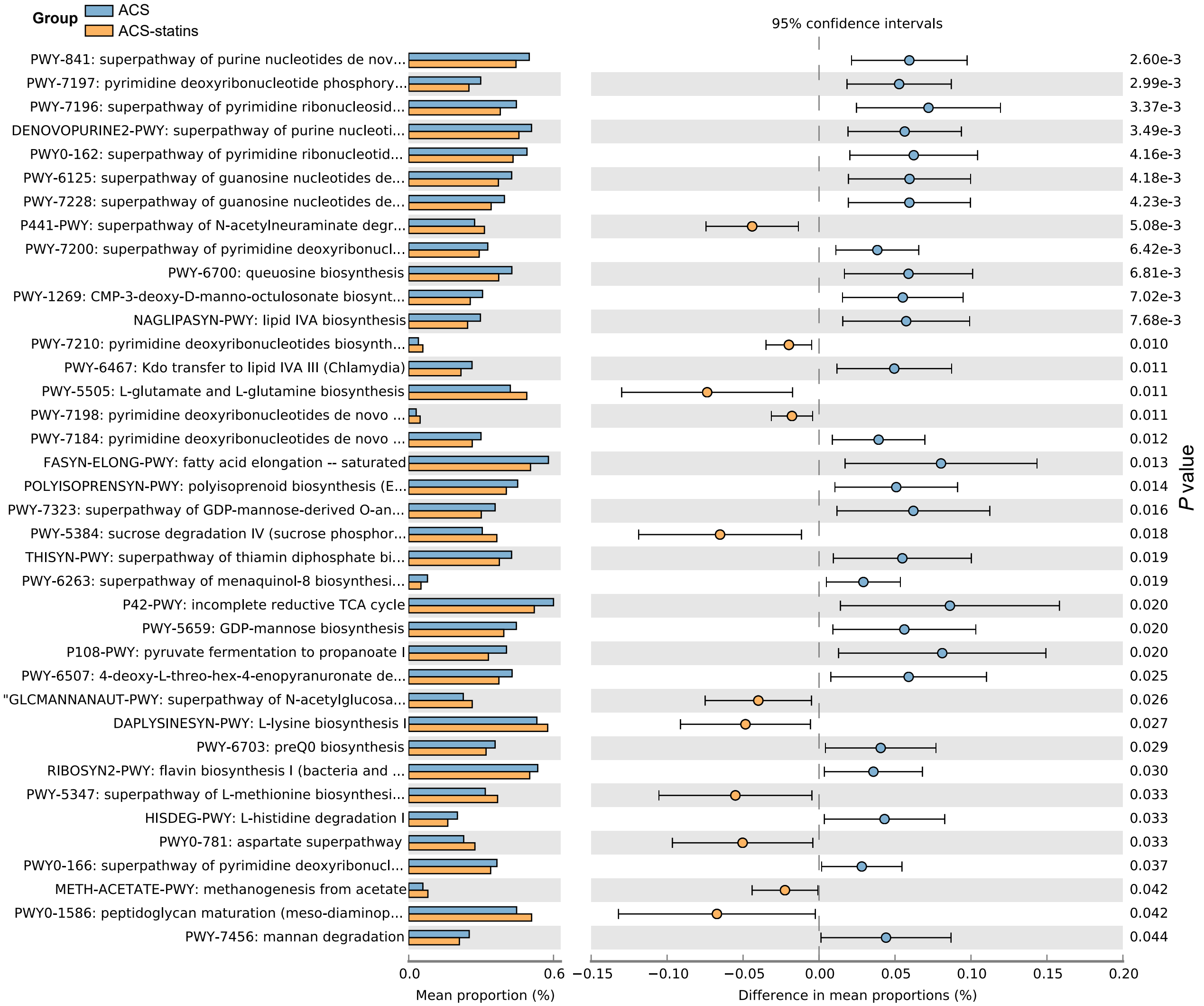

Figure S11. Differential pathways between the ACS and ACS-statins groups predicted by PICRUSt2.

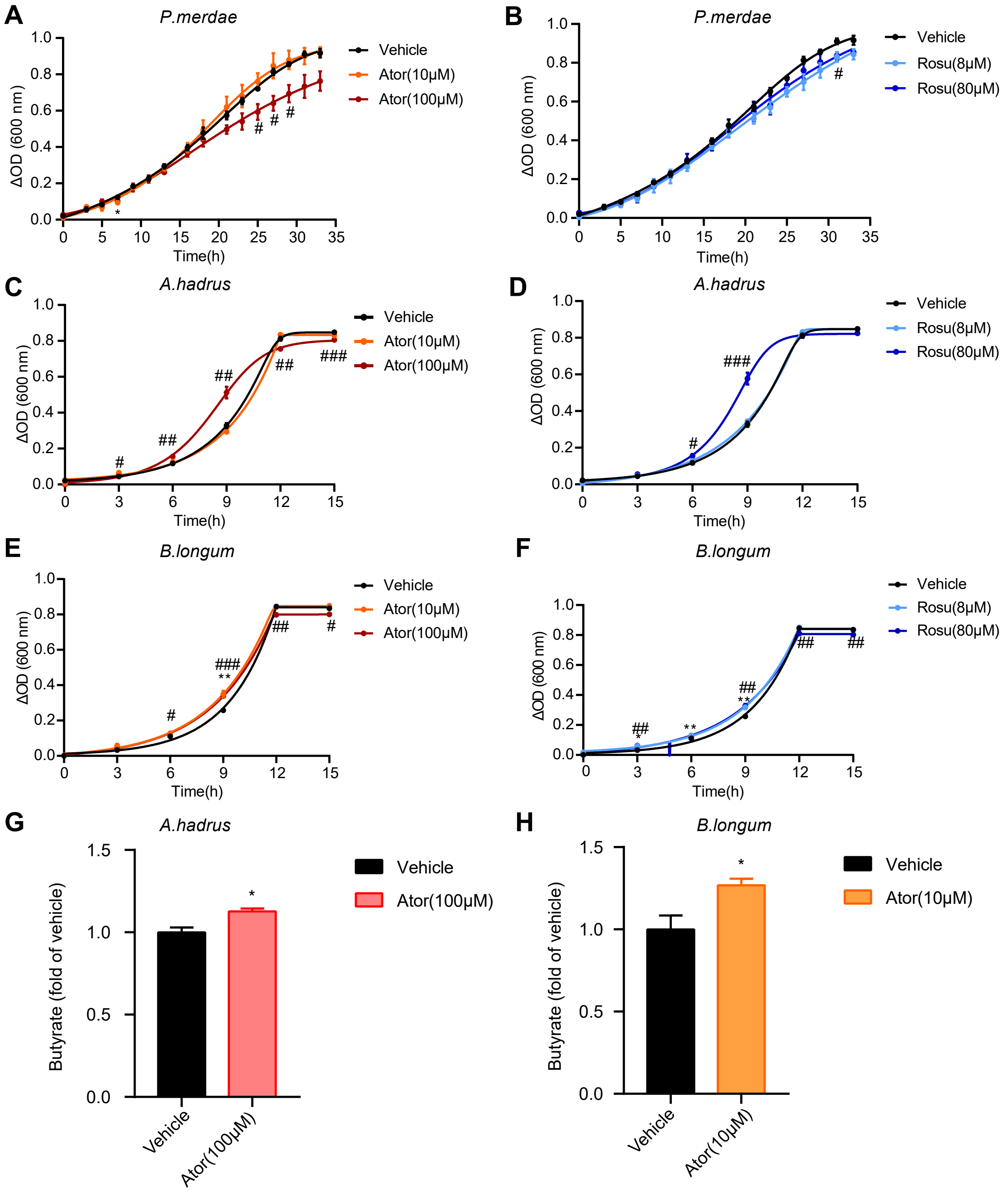

Figure S12. Growth curves of strains and content of butyrate. (**A-B**) *Parabacteroides merdae*. N = 3 per group. (**E-F**) *Anaerostipes hadrus*. N = 4 per group. (**C-D**) *Bifidobacterium longum subsp. longum*. N = 4 per group. Mean ± standard error of the mean (SEM). Student's t test. The significance between lower concentration and vehicle is presented by *. The significance between higher concentration and vehicle is presented by #. * or # $P < 0.05$, ** or ## $P < 0.01$, *** or ### $P < 0.001$. (**G-H**) The content of butyrate in *A.hadrus* or *B.longum* treated with and without statin in vitro. N = 3 per group. Student's t test. * $P < 0.05$. Ator = atorvastatin, Rosu = rosuvastatin.

Supplementary methods

Definitions of ACS subtypes

Unstable angina (UA): Defined as a normal measurement of cardiac troponin and with at least one of the following criteria: prolonged (>20 min) angina pain at rest; new onset angina (Class II or III according to the Classification of the Canadian Cardiovascular Society [1]); recent destabilization of previously stable angina with at least Canadian Cardiovascular Society Class III angina characteristics (crescendo angina); or post-myocardial infarction angina [2].

Myocardial infarction (MI): Defined as a rise and/or fall of cardiac troponin (cTnI) with at least one value above the 99th percentile upper reference limit (URL) and with at least one of the following: (1) symptoms of ischemia; (2) new or presumed new significant ST-segment-T (ST-T) wave changes or new left bundle branch block (LBBB); (3) development of pathological Q waves in the electrocardiogram; (4) imaging evidence of new loss of viable myocardium or new regional wall motion abnormality; and (5) identification of an intracoronary thrombus by angiography or autopsy [3].

ST-elevation myocardial infarction (STEMI): Conforming to the definition of MI and persistent ST elevation, which is defined as new ST elevation at the J point in at least 2 contiguous leads of 2 mm (0.2 mV) in men or 1.5 mm (0.15 mV) in women in leads V2–V3 and/or of 1 mm (0.1mV) in other contiguous chest leads or the limb leads [4].

Non-ST-elevation myocardial infarction (NSTEMI): Conforming to the definition of MI and rare ST-segment elevation upon ECG.

Statistical analysis of baseline characteristics

The Shapiro-Wilk test was employed to determine the normality of continuous data. Continuous normally distributed data are presented as the mean ± standard deviation (SD). Continuous nonnormally distributed data are presented as the median with interquartile range (IQR). Categorical variables are presented as counts and percentages. For difference comparison of clinical characteristics among the three groups, one-way

analysis of variance (ANOVA) was employed in cases of continuous normally distributed data. Bonferroni test was applied for post hoc comparisons in cases of equal variance, and Tamhane test was applied in cases of unequal variance. Kruskal-Wallis H-test was applied for continuous data that were not normally distributed among three groups, and Mann-Whitney *U* test was applied for this kind of data between two groups. Categorical variables were compared by the $\chi 2$ test or Fisher's exact test (in case of at least one expectation count < 5). The above analyses were performed using SPSS Statistics software, version 24.0 (SPSS Inc., Chicago, IL, USA) and a $P < 0.05$ was considered statistically significant.

References

1. Campeau L. Letter: Grading of angina pectoris. Circulation. 1976; 54: 522-3.
2. Authors/Task Force M, Hamm CW, Bassand J-P, Agewall S, Bax J, Boersma E, et al. ESC Guidelines for the management of acute coronary syndromes in patients presenting without persistent ST-segment elevation: The Task Force for the management of acute coronary syndromes (ACS) in patients presenting without persistent ST-segment elevation of the European Society of Cardiology (ESC). Eur Heart J. 2011; 32: 2999-3054.
3. Thygesen K, Alpert JS, Jaffe AS, Simoons ML, Chaitman BR, White HD, et al. Third universal definition of myocardial infarction. Eur Heart J. 2012; 33: 2551-67.
4. O'Gara PT, Kushner FG, Ascheim DD, Casey DE, Chung MK, Lemos JAd, et al. 2013 ACCF/AHA Guideline for the Management of ST-Elevation Myocardial Infarction. 2013; 127: e362-e425.
